# Biomass encounter rates limit the size scaling of feeding interactions

**DOI:** 10.1111/ele.13380

**Published:** 2019-08-21

**Authors:** Daniel Barrios‐O’Neill, Ruth Kelly, Mark C. Emmerson

**Affiliations:** ^1^ Environment and Sustainability Institute, Penryn Campus University of Exeter Penryn, Cornwall TR10 9EZ UK; ^2^ School of Biological Sciences and Institute of Global Food Security Queen’s University Belfast 19 Chlorine Gardens Belfast BT9 5DL Northern Ireland; ^3^ School of Natural Sciences Trinity College Dublin Dublin 2 Ireland

**Keywords:** Agent‐based modelling, body size, consumer–resource interactions, feeding rates, functional response, marine benthos

## Abstract

The rate that consumers encounter resources in space necessarily limits the strength of feeding interactions that shape ecosystems. To explore the link between encounters and feeding, we first compiled the largest available dataset of interactions in the marine benthos by extracting data from published studies and generating new data. These data indicate that the size‐scaling of feeding interactions varies among consumer groups using different strategies (passive or active) to encounter different resource types (mobile or static), with filter feeders exhibiting the weakest feeding interactions. Next, we used these data to develop an agent‐based model of resource biomass encounter rates, underpinned by consumer encounter strategy and resource biomass density. Our model demonstrates that passive strategies for encountering small, dispersed resources limits biomass encounter rates, necessarily limiting the strength of feeding interactions. Our model is based on generalisable assumptions, providing a framework to assess encounter‐based drivers of consumption and coexistence across systems.

## Introduction

Feeding interactions are fundamental to ecology, and consequential to everything from the survival and growth of individuals (Toscano & Griffen 2014), to the stability of populations (Williams & Martinez 2004), and the structure and function of whole ecosystems (Estes *et al.* 1998; Barrios‐O’Neill *et al.* 2017). To some degree, therefore, the utility of ecological science hinges on its capacity to accurately characterise feeding interactions, and to understand how the strength of these interactions is modified by intrinsic factors such as body mass (Rall *et al.* 2012; Brose *et al.* 2019), and extrinsic factors such as environmental complexity (Barrios‐O’Neill *et al.* 2016).

Well‐characterised feeding interactions combine estimates of the rate at which consumers encounter and capture resources in their environment, and estimates of the time required to handle those resources. These limiting parameters are universal to all consumers (Lafferty *et al.* 2015) and together constitute the functional response, determining feeding rates along gradients of resource density (Solomon 1949; Holling 1959). Gradients of resource density matter, because where resources are scarce – a situation typical of field conditions (Pawar *et al.* 2012) – feeding is limited by encounters rather than handling times. Moreover, for many consumers, encountering, capturing and handling resources are not mutually exclusive activities (Farnsworth & Illius 1998; Jeschke *et al.* 2004) meaning that feeding interactions can be encounter‐limited even as resource abundance increases. Thus, understanding the mechanisms that underpin and modify encounter rates is central to the wider project of characterising feeding interactions in ecology.

**Figure 1:**
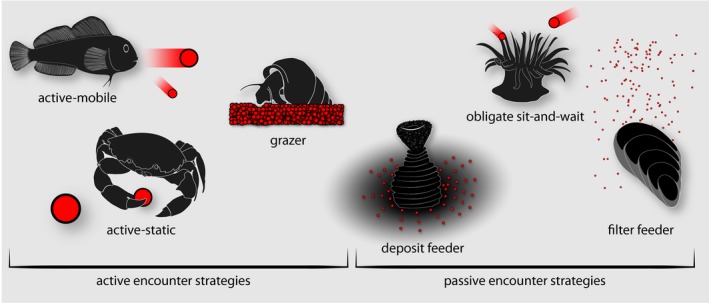
Resource encounter strategies employed by benthic consumers can be fundamentally split into two modes: consumers that actively search to encounter resources and consumers that passively encounter resources. Resources are shown in red. Further divisions can be formalised according to the type of resource. The six divisions here encompass strategies contained in the dataset and are not exhaustive.

The observation that resource consumption is ultimately driven by metabolic demand has afforded body size and its correlates a central place in models of feeding interactions (Brown *et al.* 2004; Kalinkat *et al.* 2013). Despite this centrality, body size per se is necessary but insufficient to provide a complete understanding of feeding interactions (Rall *et al.* 2011; Kalinoski & Delong 2016). For example, the dimensionality and complexity of consumer search space systematically modifies interactions (Pawar *et al.* 2012; Barrios‐O’Neill *et al.* 2015, 2016), suggesting the rate of resource biomass encounter is a key generalisable constraint. This constraint can be interpreted as the consequence of: (1) resource biomass density in the local environment, resulting from the type of resource (Damuth 1981), and also from ecological contexts (Rizzuto *et al.* 2018), including disturbance and environmental stochasticity; (2) strategies that consumers adopt to optimise encounters, such as active searching versus sit‐and‐wait tactics (Fig. 1) (Vucic‐Pestic *et al.* 2011) and; (3) the volume or area of the consumer’s search space. Given any of (1–3), large deviations from body size‐dependent predictions of feeding rates are both possible and mechanistically interpretable, provided sufficient information on strategy and context. The where and why of these deviations represents a clear knowledge gap that warrants scrutiny.

Developing a better understanding of the encounter‐feeding link is contingent on the availability of well‐characterised feeding interaction data, and such data are relatively scarce: global meta‐analyses of functional responses contain in the order of hundreds of feeding interactions across all biomes (Pawar *et al.* 2012; Rall *et al.* 2012; Li *et al.* 2018). Further, data are unevenly distributed across biomes, taxonomic groups and functional groups. Marine consumers are notably underrepresented, and the marine benthos in particular comprises only 5–10% of the largest meta‐analyses – an observation contrasting the fact that marine benthic habitats are among the most spatially extensive on the planet (Dawson 2012), hosting *c*. 94% of total marine biodiversity (May 1994). Moreover, the marine benthos includes consumers that adopt active (Fig. 1, left side) and passive (Fig. 1, right side) encounter strategies for mobile or static resources – and do so in a de facto two‐dimensional plane (that is on, in or parallel to the seabed) – making the marine benthos an ideal study system to explore the encounter‐feeding link.

Here, we compile the largest available dataset of marine benthic consumer functional responses by combining reanalysed data from 37 published studies with new experimental data. Our dataset contains 185 estimates of consumer capture and maximum feeding rates across 8 orders of magnitude in consumer body mass, and includes actively searching and sit‐and‐wait predators, grazers, filter feeders and deposit feeders. Our expectations are that passive encounter strategies and lower resource biomass densities should correlate with lower encounter and feeding rates. We analyse this dataset to identify body mass and encounter strategy dependencies of feeding rates, and show that filter feeders have lower feeding rates than other groups. Using resource body mass and abundance relations established from this data, we then develop an empirically parameterised agent‐based model of biomass encounters to demonstrate how ecological context (i.e. the type, size and biomass density of resource) combines with consumer encounter strategy (passive or active) to place an upper limit on encounter rates for filter feeders that is substantially lower than the upper limit of other groups.

## Materials and methods

### Data mining

Data was compiled by searching Google Scholar on 03/07/2017 using the following term: ‘*functional response’ OR ‘feeding rate’ OR ‘prey density’ OR ‘resource density’* (where the *OR* argument requires at least one of the four exact phrases). Search results were manually pre‐sifted for benthic and demersal marine or euryhaline taxa (yielding 33 studies) and bibliographies were searched for additional studies meeting these criteria (4 further studies) (refer to Table S1 for details of taxa and Table S2 for published data sources). We subsequently examined all studies for the following quantities: (1) consumer and resource body mass (wet mass in g); (2) trial temperature (°C), and; (3) trial arena footprint or volume (m^2^ or m^3^). Where estimates for (1) were not directly available, we used taxon‐specific length‐mass regressions or wet–dry conversion factors from the literature to estimate wet masses (all further references to consumer or resource body masses are wet masses unless otherwise stated, refer to Table S3 for specific conversion factors). Studies were discarded from the final dataset (Table S1) where any of (1–3) could not be obtained directly or estimated. Raw resource consumption data were preferentially obtained by extracting data from tables and text in papers and online repositories, or by contacting authors directly. Where this was not possible, we used DataThief III (v 1.7) to digitise data from figures.

### Supplementary experiments

We supplemented data extracted from the literature (154 responses) with new experimental data (31 responses). Each species pair that we established responses for was absent from the literature (according to our search criteria) at the time of undertaking the experimental work (Table S1, source 1 vs. sources 2‐38). Locally abundant consumers (e.g. *Carcinus maenas*) and resources (e.g. *Mytilus edulis*) allowed for multiple size treatments for some pairs (Table S4) and we also sought to generate data for undocumented functional groups [Actinaria as obligate sit‐and‐wait predators (Fig. 1)] and underrepresented taxa (Echinodermata: two existing responses, Table S1, source 31). Refer to section 2 and Table S4 of the supplementary information for full experimental methods.

### Functional response model selection and fitting

All statistical analyses were undertaken in R version 3.3.3 (R Core Team 2017).

The rate at which a consumer feeds depends on the density of available resources distributed over a surface or through a volume. The relationship between resource density and consumption is the functional response, and can be generalised as (Holling 1959; Real 1977):

(1) $$N_{e}=\frac{bN^{q+1}}{1+bhN^{q+1}},$$

where *N_e_* is the *per capita* rate of resource consumption [individuals s^−1^]; *b* is the capture rate or search coefficient of the consumer [individuals m^2^ s^−1^ or m^3^ s^−1^]; *N* is the resource density [individuals m^2^ or m^3^]; *h* is the time [s] required to subjugate and ingest a resource unit; *q* is the [dimensionless] scaling exponent, defining the extent to which the functional response departs from a hyperbola (type II) towards a sigmoidal (type III) form. Eqn (1) assumes that resource density remains constant, and this assumption is violated in experiments that do not replace resources as they are consumed. A modified version of eqn (1) provides a practical solution to this usually unavoidable violation (Rogers 1972):

(2) $$N_{e}=N_{0}\left(1-\exp\left(bN_{0}^{q+1}\left(hN_{e}-T\right)\right)\right),$$

where *N_e_* is the number of resources eaten, *N*_0_ is the initial resource density, *T* is experimental time, and other parameters are as for eqn (1). For all consumer–resource pairs across the dataset, we used maximum likelihood (Bolker 2010) to fit 4 versions of eqn (2) to consumption data, reflecting four potential response types: (1) a linear type I functional response (*sensu* Lotka–Volterra, *q* and *h* fixed at 0); (2) a hyperbolic type II functional response (*q* fixed at 0); (3) a sigmoidal type III functional response (*q* fixed at 1), and; (4) a general model, allowing all parameters to vary.

We selected the best functional response model of the (1–4) set by comparing small *n* corrected Akaike’s Information Criterion scores [AIC_c_ in the R package MuMIn (Barton 2018)] from the fitted model set. We selected the lowest scoring model unless two or more models were competitive (ΔAIC_c_ < 2), in which case we selected the most appropriate model by examining the relationship between resource density and proportional resource consumption (Juliano 2001) among competitive models. Specifically, as resource density increases: (1) constant proportional consumption is diagnostic of a type I functional response; (2) decreasing proportional consumption is diagnostic of a type II functional response, and; (3) increasing then decreasing proportional consumption is diagnostic of a type III functional response (Pritchard *et al.* 2017). We selected the most appropriate model from competing sets by fitting locally‐weighted regressions to proportional consumption data to directly test for trends (1–3) indicative of functional response type.

### Meta‐analysis

Here, we develop a categorisation of consumers according to encounter strategy (Fig. 1) based on a combination of existing approaches. First we assume that consumers either actively encounter (i.e. search for) resources (Fig. 1, left side, velocity > 0) or passively encounter resources (Fig. 1, right side, velocity = 0) (Pawar *et al.* 2012). Among active searchers, potential resources can be mobile (an active‐mobile interaction) or static (an active‐static interaction). Unlike Pawar *et al. *(2012) we define grazing as distinct from an active‐static interaction where encounter, capture and handling activites are not mutually exclusive (Spalinger & Hobbs 1992), and where partial consumption of large static resources or composite resources occurs (e.g. macroalgae or biofilms). Passive searchers are differentiated from active searchers where encounters depend primarily on movement of resources in space. Among passive searchers, we adopt a stricter definition of filter feeders than Jeschke *et al. *(2004) by differentiating those consumers that search for in‐ or on‐seabed resources (deposit feeders), and those consumers searching for resources that are relatively large (obligate sit‐and‐wait predators).

Metabolic theory leads to expectations of power law relationships between consumer body mass and consumer capture coefficients or handling times (*b* and *h*, eqn 1)(Rall *et al.* 2012; Li *et al.* 2018). Therefore, we log*_e_* transformed mass, temperature and feeding parameters prior to analysis. Because our data include functional responses where *q *> 0 (implying an increase in capture rates with resource density, i.e. captures ∝ *N^q^* ^+1^), we use slopes at the half saturation ($\frac{0.5}{h}$) resource density to include functional responses with *q *> 0 in the analysis of capture rates (Englund *et al.* 2011; Rall *et al.* 2012).

Two maximal random intercept mixed‐effects models with capture rates (g m^2 or 3 −1^ d^−1^) and maximum feeding rates (g d^−1^) as standardised dependent variables were fitted using the R package lme4 (Bates *et al.* 2015) (refer to section 3 of the supplement for model code). We assume here that taxonomic grouping within encounter strategies will affect the variance of the response and, therefore, taxonomic grouping is treated as a random effect in our model structure. Rather than imposing an *a priori* Arrhenius temperature correction on response parameters (Rall *et al.* 2012), temperature was also treated as a fixed effect such that the maximal model structure for either response variable included all two‐way interactions between metabolic predictors (temperature, consumer mass, resource mass) and encounter strategy as a non‐interacting categorical predictor.

Given the starting maximal model structure (section S3), 36 combinations of fixed‐effects (including the null) are possible. For both capture and maximum feeding rate models, we selected the most appropriate model, i.e. the model with the lowest AIC_c_ score, using the dredge function in the R package MuMIn (Barton 2018) (Table S5). Because some encounter strategies were extremely data‐poor, we focus our analysis on active encounter consumers foraging on static and mobile resources, and filter feeders – together constituting 92% of the data – but retain other strategies in plots for qualitative comparisons. Finally, because our functional response model selection process resulted in an atypical distribution for scaling exponents (positive non‐integer values combined with a predominance of 0s and 1s), we opted to non‐parametrically bootstrap (*n* = 2000) mean exponent values between groups to allow for useful comparisons of this parameter.

### An agent‐based model of biomass encounters

**Figure 2:**
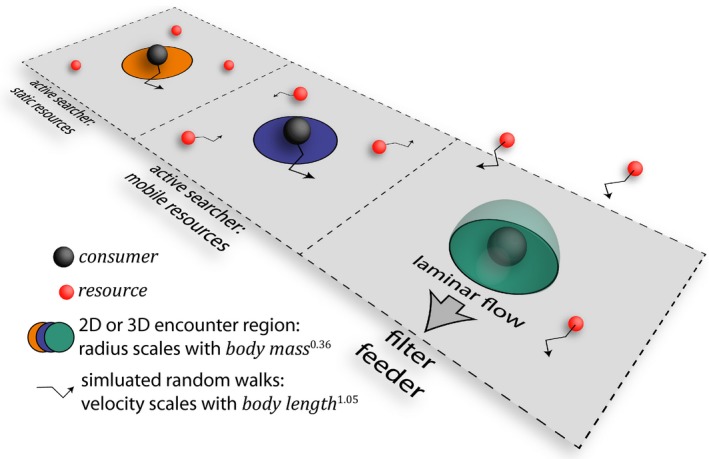
Framework for an agent‐based model of biomass encounter rates. Three assumptions underpin movement and resource encounters in the model: (1) benthic consumers have either discoid (orange and blue) or hemispheric (green) encounter regions that scale with body mass; (2) consumers and resources that move do so via random walks at a velocity that scales with body length, and; (3) planktonic resources are also subject to unidirectional laminar flow imposed on their random walks.

Here, we develop an empirically parametrised Agent‐Based Model (ABM) in Netlogo (6.0.4) to predict maximal resource biomass encounter rates for consumers as a result of our three focal encounter strategies, and the scaling of the following variables with consumer or resource body mass: (1) consumer body velocity; (2) consumer encounter region size; (3) resource body velocity; (4) the unitary density of resources in space, and; (v) the unitary mass of resources. For (1) and (3) we refitted velocity data from Vogel (2008) for the range of body masses in our dataset (Table S1 and Fig. S1) yielding:

(3) $$V=2.67\cdot l^{1.05},$$

where *V* is consumer or resource velocity (m s^−1^) and *l* is consumer or resource body length (m). For (2) we define a benthic consumer’s encounter region as a discoid (2D) or hemisphere (3D) limited by that consumer’s reaction distance (i.e. the radius of the *n*‐dimensional encounter region) which scales with consumer mass (*m* ^0.36^)(Pawar *et al.* 2012) (Fig. 2). Alternatively, for filter feeders in benthic habitats, the clearance rate (i.e. litres of water cleared of resources each hour) can be used to define a smaller hemispheric encounter region (eqn S3 and Fig. S2) and we include this as a supplementary sensitivity analysis (Fig. S5). We assume here that demersal fish move predominantly in the x–y plane, that is, parallel to the seabed, and thus actively encounter resources in de facto 2D, whilst filter feeders encounter resources passively in 3D because their resources move throughout the water column. For each of the three focal strategies (filter feeding, active‐mobile and active‐static), we estimate the scaling of unitary resource density (4) and mass (5) as a function of consumer mass using OLS regression with a range of coefficients and exponents specific to each strategy (Figs S3 and S4, Table S4). We focus on minimum resource density because, when resources are scarce, consumption is limited by encounters rather than handling times (de Roos *et al.* 1991). Finally, we assume that consumers and resources that move do so via simulated random walks (Bartumeus *et al.* 2005) and, further, that planktonic resources are also subject to a unidirectional laminar current running parallel to the seabed at 0.1 m s^−1^ ± 0.01, which reflects an environmentally‐relevant velocity that is within the optimal range for filter feeding (Ackerman 1999; Widdows *et al.* 2002).

## Results

### Data

Our dataset contains 185 consumer‐resource pairs, with consumer masses spanning eight orders of magnitude (10^−6^–10^2^ g) and resource masses spanning twelve (10^−11^–10^1^ g). The dataset includes crustaceans (*n* = 86), bony and cartilaginous fish (*n* = 34), tunicates (*n* = 19), bivalve molluscs (*n* = 16), cnidarians (*n* = 8), gastropod molluscs (*n* = 7), echinoderms (*n* = 7), polychaetes (*n* = 4) and bryozoans (*n* = 4). Among these groups, active encounter strategies (Fig. 1, left side) are dominated by active searchers for static resources (*n* = 70) and active searchers for mobile resources (*n* = 61), whilst passive strategies (Fig. 1, right side) are dominated by filter feeders (*n* = 40).

## Meta‐analysis

**Figure 3:**
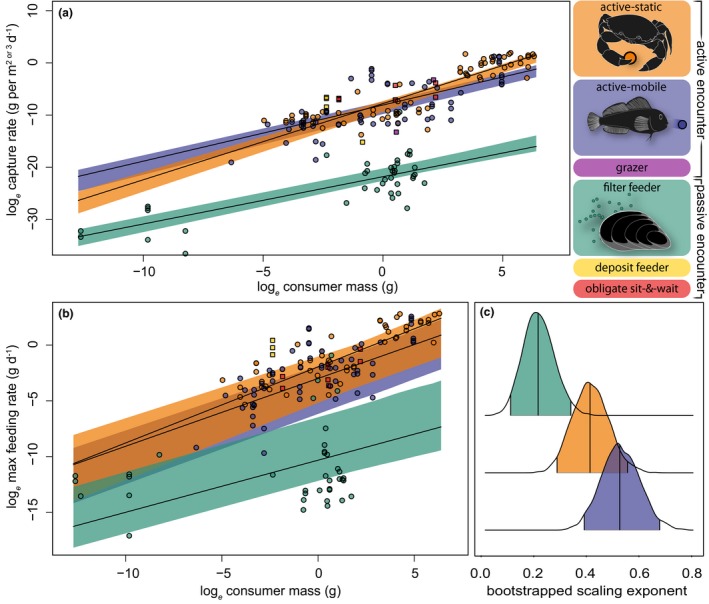
Consumer size scaling of resource capture rates (a) (*n* = 185) and maximum feeding rates (b) (*n* = 148) with encounter strategy, and (c) scaling exponents indicating resource density‐dependence of capture rates among strategies. Solid lines in (a) and (b) are predictions from lowest scoring mixed effects models (Fig. 4); shaded areas are bootstrapped 95% confidence intervals (a–c). Capture and maximum feeding rate data points from small‐*n* groups are shown but not modelled (all square points in (a) and (b), non‐pictogram colour blocks in key). Ridge plots (c) produced using the R package ggridge (Wilke 2018).

**Figure 4:**
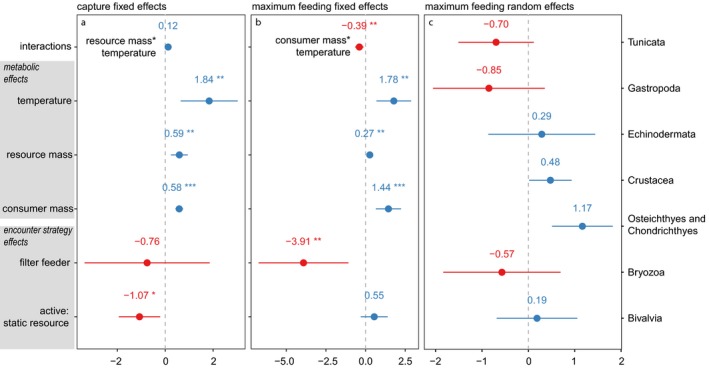
Mixed effects model predictions for lowest scoring capture and maximum feeding rate models. Fixed effects structures for these models are: (a) *capture* ~ *consumer mass* + *resource mass* * *temperature* + *encounter strategy*, and; (b) *maximum feeding* ~ *consumer mass* * *temperature* + *resource mass* + *encounter strategy.* Red points are below group intercept coefficients, blue points are above, and bars are 95% confidence intervals. In (a) and (b) stars denote significance: *p < 0.05, **p < 0.01, ***p < 0.001. Taxonomic grouping random effects for maximum feeding rates are shown in (c). Taxonomic grouping random effects for capture rates all intersect with the group intercept. Plots produced with the R package sjPlot (Lüdecke 2017).

Increased consumer mass, resource mass and temperature all drive higher capture rates across encounter strategies (Figs 3a and 4a), and capture rates are best explained by a mixed‐effects model including each of these fixed effects, with the lowest scoring model including a resource mass‐temperature interaction (Fig. 4a, AIC_c_: null model (intercept only) = 1073.0; global = 784.7; lowest scoring model = 784.0). We find that encounter strategy has a clear association with the scaling of capture rates (Fig. 3a). Specifically, filter feeders capture significantly less resource biomass than active‐mobile or active‐static consumers for a given consumer mass (Fig. 3a: green non‐overlapping 95% CIs), and these differences between encounter strategies are driven primarily by differences in resource size (Fig. 4a). Further, data points for encounter strategies excluded from analysis (obligate sit‐and‐wait predators (*n* = 8), deposit feeders (*n* = 4) and grazers (*n* = 2)) are also all above the upper CI bound for filter feeders (Fig. 3a: square points). We also find that the largest active‐static consumers capture more resource biomass than equivalently sized active‐mobile consumers (Fig. 3a: non‐overlapping orange and blue 95% CIs). As with capture rates, increased consumer size, resource size and temperature all drive higher maximum feeding rates (Figs 3b and 4b). Maximum feeding rates are also best explained by the same set of fixed effects, and the lowest scoring model also includes a consumer mass–temperature interaction (Fig. 4b, AIC_c_: intercept only = 740.5; global = 661.2; lowest scoring model = 657.2). Filter feeders exhibit lower maximum feeding rates at larger consumer masses than other groups (Figure 3b: non‐overlapping green 95% CIs at larger consumer masses). Unlike capture rates, however, these lower maximum feeding rates are driven not just by resource size and metabolic predictors but also by the filter feeding strategy per se (Fig. 4b). Random taxonomic effects do not explain variance in capture rates but do have explanatory power for maximum feeding rates (Fig. 4c). Finally, filter feeder capture rates are the least resource density dependent (smallest *q* (eqn 1): Fig. 3c green) while active‐mobile capture rates are the most resource density dependent (largest *q*: Fig. 3c blue).

### Agent‐based model predictions

**Figure 5:**
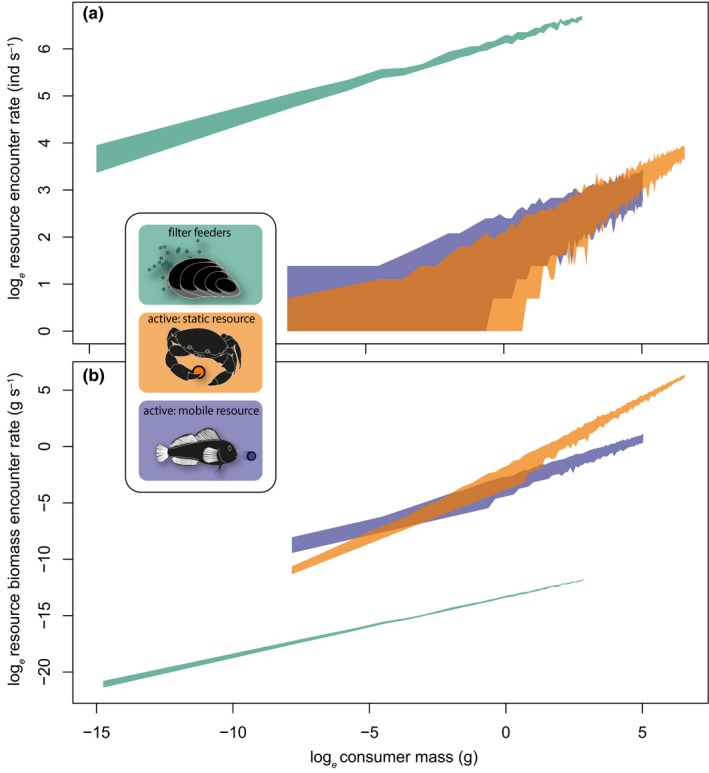
Agent‐based model outputs of resource encounter rates among encounter strategies (inset) for (a) resource units in individuals s^−1^ and (b) resource biomass in g s^−1^. Shaded areas are interpolated 0.025 (lower) and 0.975 (upper) quantiles (*n* = 1000) of encounters along the consumer mass axis. The model is empirically parameterised with respect to consumer masses, resource masses and resource densities (Figs S3 and S4) underpinning the meta‐analysis (Figs 3 and 4).

Our model predicts increasing unitary resource encounter rate with increasing consumer mass across all strategies (Fig. 5a), reflecting experimentally derived consumer capture rates (Fig. 3a). Notably, on account of the small and abundant nature of planktonic resources, filter feeders (Fig. 5a: green) encounter more resource units at a given mass than active‐mobile and active‐static consumers. No clear differences between active‐mobile and active‐static unitary resource encounters emerge (Fig. 5a: overlapping blue and orange quantile areas). Predicted biomass encounter rates reflect unitary encounter rates in their positive scaling with consumer mass across encounter strategies (Fig. 5b). However, biomass encounter rates are lower for filter feeders (Fig. 5b: green) than for other consumers, and this result is robust to assumptions about encounter region size (Fig. S5). Further, our model predicts that the largest active‐static consumers experience higher biomass encounter rates than equivalently sized active‐mobile consumers (Fig. 5b: non‐overlapping orange and blue quantile regions), reflecting experimentally derived capture rates (Fig. 3a: non‐overlapping orange and blue 95% CIs).

## Discussion

We have shown here that consumer encounter strategies are associated with large, non‐random differences in feeding interactions among marine benthic consumers. In particular, the low size‐specific capture and maximum feeding rates exhibited by filter feeders are previously unrecognised marcoecological features of benthic systems. Our model provides a parsimonious and generalisable explanation for this feature by highlighting the primacy of resource biomass encounter rates in limiting the strength of feeding interactions. Thus, we argue that simple assumptions about consumer encounter strategies and resource biomass could usefully refine estimates of feeding interactions in complex food webs across habitats and biomes. These encounter‐based estimates are not wholly derivable from consumer body size and its correlates (Brown *et al.* 2004; Rall *et al.* 2012) and have the potential to explain large and apparently idiosyncratic macroecological patterns of resource consumption. Because the ecological impacts of global environmental change often manifest through proximate effects on consumer–resource interactions (Cahill *et al.* 2012; Dick *et al.* 2017), resolving and understanding the consequences of these patterns should be a central and urgent objective for ecological science going forward.

Metabolic theory predicts universal body mass dependencies of many biological processes, including feeding interactions (Brown *et al.* 2004). But empirically derived feeding interaction data are notoriously complex (Pawar *et al.* 2012; Kalinkat *et al.* 2013; Barrios‐O’Neill *et al.* 2015, 2016; Kalinoski & Delong 2016), and ecologists are also aware that taxonomic identity maps onto these universal trends (Rall *et al.* 2011; Kalinoski & Delong 2016). However, taxonomic identity should not be viewed as irreducible, because extrinsic factors such as search space dimensionality can provide alternative mechanistic explanations (Pawar *et al.* 2012). Our data demonstrate that higher‐level taxonomic groups with equivalent encounter strategies can be amalgamated (Fig. 4a), though these taxonomic groupings continue to explain variance in feeding rates (Fig. 4b and c). Further, subsetting by taxonomic group in the first instance will preclude useful predictions where consumers switch encounter strategies (for example, switching from mobile to static resources), switch from surface to volume foraging, or forage through habitats of varying complexity (Barrios‐O’Neill *et al.* 2015, 2016). We view each of these changes in foraging context as interpretable in terms of biomass encounter rates. For example, our data includes actively‐searching predators of the same species (Table S1) that switch between mobile and static resources, and our predictions of biomass encounter rates for these strategies (Fig. 5b: orange and blue regions) reflect the increased capture rates associated with larger consumers foraging for more densely concentrated static resources (Fig. 3a orange). This active‐static difference is subtle, but potentially important in providing a mechanistic basis for understanding ontogenetic shifts in diet (Perez & Bellwood 1988).

The strategy‐based differences we observe in encounter rates may necessitate in‐kind differences in metabolic rates, basal or otherwise, or may instead be mitigated by in‐kind differences in the proportion of time consumers spend foraging (Rizzuto *et al.* 2018). Alternatively, it is conceivable that the non‐random space use and movement patterns exhibited by many consumers and resources result in misleading estimates of encounter and capture rates among strategies (Uiterwall *et al.* 2018). It is also apparent that our phenomenological approximation of filter feeding detection regions do not capture the range of encounter mechanisms available to consumers defined as filter feeders (Jeschke *et al.* 2004). However, our results do question the presupposition that the linear functional responses unique to filter feeders are adaptively significant because they allow for higher feeding rates (Jeschke *et al.* 2004; Denny 2014). Not all filter feeding strategies should be treated equally in this regard, because many pelagic filter feeders are active searchers—ranging from large cetaceans to *Daphnia*—moving through their environment to locate and exploit high concentrations of resources (Jensen *et al.* 2001; Hazen *et al.* 2015). Although many passively searching benthic filter feeders move, they move only for limited time periods at small spatial scales, to optimise their chances of survival rather than to track resources (Van De Koppel *et al.* 2008; Barrios‐O’Neill *et al.* 2017). Further, the filter‐feeding responses we consider here are predominantly nonlinear (Fig. 3b), and refined experiments are revealing more nonlinear responses in pelagic and benthic filter feeders alike (Sarnelle & Wilson 2008; Sarnelle *et al.* 2015), suggesting that response linearity is not fundamental to the adaptive significance of filter feeding.

The distinction between active and passive searching serves to illustrate the wider point that encounters between consumers and resources are necessary and consequential to any feeding interactions that follow. Although ecologists have known this for decades, the fact that some fundamental characteristics of consumer search space have only recently been linked to encounters and feeding (Pawar *et al.* 2012) suggests there is much opportunity for further progress. The link between encounters and feeding therefore matters, although we must stress that encounter rates per se cannot directly predict feeding rates, particularly at high resource densities. Yet the concordance between capture (Fig. 3a) and maximum feeding rates (Fig. 3b) among encounter strategies implies that, at minimum, a predictive understanding of biomass encounter rates can be used to set defined upper limits to the parameters of consumption.

Feeding interactions are fundamental to ecology, because survival, growth and reproduction requires that all organisms consume resources of some kind. Despite decades of progress in our understanding of the mechanics and physiology underpinning feeding interactions (Stephens & Krebs 1986; Arditi & Ginzburg 2012), we argue that further developments are ultimately limited by the quantity of empirical data thus far accumulated. Our study adds new data for marine benthic consumers that are under‐represented in the literature, and highlights the scarcity of data describing well‐characterised interactions globally. We believe that two complementary programmes of work are required to address knowledge gaps going forward: the first should seek to systematise and standardise the empirical collection of data on feeding interactions across species and systems; the second should use these data to unify metabolic and non‐metabolic drivers of resource consumption in a single framework. Together, these programmes can provide ecologists with the data and tools to refine urgently required whole‐system forecasts for the future.

## Authorship

DBO designed and conducted experimental trials, extracted and refitted functional responses from the literature, and built agent‐based models. RK and DBO fitted mixed effects models. DBO, MCE and RK wrote the manuscript.

## Data Availability

Raw data from experimental trials is available at https://github.com/DBarriosONeill/biomass-encounter-trial-data and at https://doi.org/10.5281/zenodo.3357928.
