# Supplementary material for: Biomass encounter rates limit the size scaling of feeding interactions
Source: Ecol Lett. 2019 Aug 21;22(11):1870–8. doi: 10.1111/ele.13380 (PMC6852157; doi:10.1111/ele.13380)
Supplement: Supplementary file 1 [file ELE-22-1870-s001.pdf]

Supplementary information for: Biomass encounter rates limit the size scaling of feeding interactions

Daniel Barrios-O'Neill [*a, b], Ruth Kelly [c] and Mark C. Emmerson [b]

[a] Environment and Sustainability Institute, Penryn Campus, University of Exeter, Penryn, Cornwall, TR10 9EZ, UK

[b] School of Biological Sciences and Institute of Global Food Security, Queen's University Belfast, 19 Chlorine Gardens, Belfast, BT9 5DL, Northern Ireland m.emmerson@qub.ac.uk

[c] School of Natural Sciences, Trinity College Dublin, Dublin 2, Ireland

[*] Corresponding author: d.barrios-oneill@exeter.ac.uk / +447846118860

CONTENTS

1. META-ANALYSIS DATA (pages 2-18)

2. SUPPLEMENTARY EXPERIMENTS (pages 19-21)

3. MIXED EFFECTS MODELS (pages 22-24)

4. AGENT-BASED MODEL PARAMETERISATION (pages 25-28)

5. AGENT-BASED MODEL CODE (pages 29-31)

6. FILTER FEEDER DETECTION REGION SENSITIVITY ANALYSIS (page 32)

1. META-ANALYSIS DATA

Table S1 Meta-analysis data table. Fitted capture coefficients and handling times (eqn 2) are in units of g per m$^{2 \text{ or } 3}$ d^{-1} and d, respectively. Consumer-resource colour blocks are as per categories in Fig 1: (blue) active-mobile; (orange) active-static; (green) filter feeder; (red) obligate sit-and-wait; (purple) grazer, and; (yellow) deposit feeder. Data sources column is split as follows: consumption data / consumer mass / resource mass, with numbers referring to data sources in Tables S2 (consumption) and S3 (conversion factors as required for consumers and resources—identical numbers to consumption source indicate wet masses were given in the study). Raw consumption data from experimental trials available at: https://github.com/DBarriosONeill/biomass-encounter-trial-data

data sources	consumer→ resource	consumer mass (g)	resource mass (g)	volume (cm^3)	footprint (cm^2)	temperature (°C)	time (d)	minimum resource density (m$^{2 \text{ or } 3}$)	maximum resource density (m$^{2 \text{ or } 3}$)	capture coefficient (g per m$^{2\text{-}3}$ d^{-1})	scaling exponent	handling time (d)
1/1/1	*Carcinus maenas→ Echinogammarus marinus*	6.86E+00	4.70E-02	3424.18	407.64	7.91	1.00	4.91E+01	7.85E+02	1.10E-03	0.95	6.08E-02
1/1/1	*Lipophrys pholis→ Echinogammarus marinus*	3.36E+00	4.50E-02	3424.18	407.64	7.91	1.00	4.91E+01	7.85E+02	1.64E-03	1.00	1.81E-01
1/1/1	*Lipophrys pholis→ Echinogammarus marinus*	9.59E+00	4.90E-02	3424.18	407.64	7.99	1.00	4.91E+01	7.85E+02	8.37E-03	0.00	4.44E-02
1/1/1	*Carcinus maenas→ Echinogammarus marinus*	2.58E+01	5.50E-02	11225.10	905.25	7.85	1.00	2.21E+01	3.53E+02	3.28E-03	0.00	4.31E-02
1/1/1	*Pholis gunnellus→ Echinogammarus marinus*	5.71E+00	5.00E-02	3424.18	407.64	8.03	1.00	4.91E+01	3.93E+02	2.22E-03	0.45	1.82E-01
1/1/1	*Carcinus maenas→ Mytilus edulis*	1.03E+02	1.37E+01	31524.00	1970.25	8.00	0.66	2.03E+01	1.62E+02	4.20E-01	1.00	4.76E-01
1/1/1	*Carcinus maenas→ Mytilus edulis*	9.40E+01	6.52E+00	31524.00	1970.25	8.00	0.66	2.03E+01	1.62E+02	3.01E+00	0.00	1.50E-01
1/1/1	*Carcinus maenas→ Mytilus edulis*	9.66E+01	1.08E+00	31524.00	1970.25	8.00	0.66	2.03E+01	3.25E+02	5.03E-01	0.25	1.98E-02
1/1/1	*Carcinus maenas→ Mytilus edulis*	6.69E+01	1.37E+01	31524.00	1970.25	7.92	0.66	2.03E+01	1.62E+02	2.16E+00	0.00	5.22E-01
1/1/1	*Carcinus maenas→ Mytilus edulis*	6.02E+01	6.52E+00	31524.00	1970.25	8.00	0.66	2.03E+01	1.62E+02	7.85E-02	1.00	5.35E-01
1/1/1	*Carcinus maenas→ Mytilus edulis*	6.02E+01	1.08E+00	31524.00	1970.25	8.00	0.66	2.03E+01	3.25E+02	2.89E-01	0.17	1.39E-02
1/1/1	*Carcinus maenas→ Mytilus edulis*	2.51E+01	1.37E+01	31524.00	1970.25	7.92	0.66	2.03E+01	1.62E+02	3.47E-03	1.00	1.49E+00

1/1/1	*Carcinus maenas→ Mytilus edulis*	2.53E+01	6.52E+00	31524.00	1970.25	8.00	0.66	2.03E+01	1.62E+02	3.32E-03	1.00	7.44E-01
1/1/1	*Carcinus maenas→ Mytilus edulis*	2.52E+01	1.08E+00	31524.00	1970.25	8.00	0.66	2.03E+01	3.25E+02	5.31E-03	1.00	3.94E-02
1/1/1	*Actinia equina→ Echinogammarus marinus*	1.58E-01	1.22E-01	11225.10	905.25	12.00	1.00	2.21E+01	2.76E+02	8.50E-04	0.00	0.00E+00
1/1/1	*Actinia equina→ Echinogammarus marinus*	1.67E+00	1.22E-01	11225.10	905.25	12.00	1.00	2.21E+01	2.76E+02	1.03E-03	1.09	8.89E-01
1/1/1	*Actinia equina→ Echinogammarus marinus*	8.99E+00	1.22E-01	11225.10	905.25	12.00	1.00	2.21E+01	2.76E+02	6.93E-04	1.00	1.78E-01
1/1/1	*Actinia equina→ Echinogammarus marinus*	1.67E+00	5.90E-02	11225.10	905.25	12.30	1.00	2.21E+01	2.76E+02	8.11E-04	0.00	3.27E-01
1/1/1	*Actinia equina→ Echinogammarus marinus*	1.67E+00	7.74E-03	11225.10	905.25	12.30	1.00	2.21E+01	2.76E+02	1.64E-05	1.00	1.75E-01
1/1/1	*Actinia equina→ Echinogammarus marinus*	1.58E-01	5.90E-02	11225.10	905.25	12.00	1.00	2.21E+01	2.76E+02	2.12E-05	1.00	9.87E-01
1/1/1	*Actinia equina→ Echinogammarus marinus*	8.99E+00	5.90E-02	11225.10	905.25	11.90	1.00	2.21E+01	2.76E+02	1.41E-03	0.00	2.60E-01
1/1/1	*Actinia equina→ Echinogammarus marinus*	1.58E-01	7.74E-03	11225.10	905.25	11.90	1.00	2.21E+01	2.76E+02	5.39E-05	1.00	3.74E-01
1/1/1	*Marthasterias glacialis→ Mytilus edulis*	5.48E+02	1.37E+01	134926.10	4190.00	15.22	2.75	2.39E+00	1.43E+01	7.48E-01	1.00	8.15E-01
1/1/1	*Asterias rubens→ Mytilus edulis*	9.87E+01	5.70E+00	31524.00	1970.25	15.02	2.75	5.08E+00	3.05E+01	1.31E-01	2.62	1.10E+00
1/1/1	*Asterias rubens→ Mytilus edulis*	3.88E+01	5.70E+00	11225.10	905.25	15.02	2.75	1.10E+01	4.42E+01	3.17E-01	0.00	1.73E+00
1/1/1	*Asterias rubens→ Mytilus edulis*	1.60E+02	5.70E+00	31524.00	1970.25	15.22	2.75	5.08E+00	3.05E+01	4.02E-01	0.00	1.79E-01
1/1/1	*Asterias rubens→ Mytilus edulis*	9.87E+01	1.08E+00	31524.00	1970.25	13.10	2.75	5.08E+00	2.03E+01	8.60E-02	0.00	2.92E-01
1/1/1	*Necora puber→ Mytilus edulis*	1.57E+02	3.44E+00	31524.00	1970.25	15.20	0.75	1.02E+01	1.02E+02	3.10E+00	0.00	1.14E-01
1/1/1	*Necora puber→ Mytilus edulis*	7.05E+01	4.44E+00	31524.00	1970.25	15.20	0.75	5.08E+00	1.02E+02	9.67E-01	0.00	2.74E-01
1/1/1	*Cancer pagurus→ Mytilus edulis*	2.06E+02	4.44E+00	31524.00	1970.25	13.10	0.75	1.02E+01	2.03E+02	4.33E-01	1.01	4.30E-02
1/1/1	*Cancer pagurus→ Mytilus edulis*	4.07E+02	1.20E-01	31524.00	1970.25	13.50	0.75	5.08E+00	1.02E+02	2.46E-03	1.00	9.61E-02

2/39/40	*Saduria entomon→ Monoporeia affinis*	3.86E-01	8.00E-04	60000.00	2000.00	5.70	1.00	3.00E+02	2.30E+03	4.30E-08	1.00	1.04E-01
2/39/40	*Saduria entomon→ Monoporeia affinis*	3.86E-01	3.78E-03	60000.00	2000.00	5.70	1.00	3.00E+02	2.30E+03	7.20E-08	1.00	1.59E-01
2/39/40	*Saduria entomon→ Monoporeia affinis*	3.86E-01	1.16E-02	60000.00	2000.00	5.70	1.00	3.00E+02	2.30E+03	1.41E-07	1.00	7.98E-01
2/39/40	*Saduria entomon→ Monoporeia affinis*	2.49E+00	8.00E-04	60000.00	2000.00	5.70	1.00	3.00E+02	2.30E+03	5.00E-08	1.00	1.01E-01
2/39/40	*Saduria entomon→ Monoporeia affinis*	2.49E+00	3.78E-03	60000.00	2000.00	5.70	1.00	3.00E+02	2.30E+03	8.90E-07	1.00	9.67E-02
2/39/40	*Saduria entomon→ Monoporeia affinis*	2.49E+00	1.16E-02	60000.00	2000.00	5.70	1.00	3.00E+02	2.30E+03	3.84E-07	1.16	9.05E-02
2/39/40	*Saduria entomon→ Monoporeia affinis*	7.75E+00	8.00E-04	60000.00	2000.00	5.70	1.00	3.00E+02	2.30E+03	5.54E-08	1.00	5.99E-02
2/39/40	*Saduria entomon→ Monoporeia affinis*	7.75E+00	3.78E-03	60000.00	2000.00	5.70	1.00	3.00E+02	2.30E+03	2.20E-08	1.62	4.99E-02
2/39/40	*Saduria entomon→ Monoporeia affinis*	7.75E+00	1.16E-02	60000.00	2000.00	5.70	1.00	3.00E+02	2.30E+03	1.23E-06	1.00	1.15E-01
2/39/40	*Saduria entomon→ Monoporeia affinis*	1.71E+01	8.00E-04	60000.00	2000.00	5.70	1.00	3.00E+02	2.30E+03	3.22E-08	1.00	8.98E-02
2/39/40	*Saduria entomon→ Monoporeia affinis*	1.71E+01	3.78E-03	60000.00	2000.00	5.70	1.00	3.00E+02	2.30E+03	3.74E-07	1.00	5.46E-02
2/39/40	*Saduria entomon→ Monoporeia affinis*	1.71E+01	1.16E-02	60000.00	2000.00	5.70	1.00	3.00E+02	2.30E+03	1.37E-06	1.00	6.05E-02
3/41/42	*Paralabrax clathratus→ Brachyistius frenatus*	1.28E+02	1.21E+00	5000000.00	50000.00	20.00	0.63	2.00E+00	1.20E+01	3.43E+00	0.00	1.10E-01
3/41/42	*Paralabrax clathratus→ Brachyistius frenatus*	1.28E+02	1.21E+00	5000000.00	50000.00	20.00	0.63	2.00E+00	1.20E+01	2.99E+00	0.00	1.83E-01
3/41/42	*Paralabrax clathratus→ Brachyistius frenatus*	1.28E+02	1.21E+00	5000000.00	50000.00	20.00	0.63	2.00E+00	1.20E+01	1.37E+00	0.00	9.31E-02
3/41/42	*Paralabrax clathratus→ Brachyistius frenatus*	1.28E+02	1.21E+00	5000000.00	50000.00	20.00	0.63	2.00E+00	1.20E+01	9.92E-01	0.00	1.28E-01
4/43/44	*Crangon crangon→ Macoma balthica*	4.02E-02	2.44E-05	4000.00	314.00	15.00	0.01	6.37E+01	9.71E+03	5.63E-05	0.00	1.23E-04
4/43/44	*Crangon crangon→ Macoma balthica*	4.02E-02	2.44E-05	4000.00	314.00	15.00	0.01	6.37E+01	9.94E+03	3.45E-05	0.00	1.25E-04
4/43/44	*Crangon crangon→ Macoma balthica*	4.02E-02	2.44E-05	4000.00	314.00	15.00	0.01	1.27E+02	9.94E+03	4.33E-05	0.00	2.70E-04

5/5/45	*Zoobotryon verticillatum*→ *Monochrysis lutheri*	5.46E-05	8.10E-11	200.00	200.00	24.00	0.13	2.16E+07	3.98E+08	1.94E-15	0.00	2.18E-03
5/5/45	*Zoobotryon verticillatum*→ *Cricosphaera carterae*	5.46E-05	1.03E-10	200.00	200.00	24.00	0.13	3.69E+06	1.45E+08	5.58E-13	0.00	7.43E-05
5/5/45	*Zoobotryon verticillatum*→ *Dunaliella tertiolecta*	5.46E-05	2.57E-10	200.00	200.00	24.00	0.13	2.18E+07	4.39E+08	1.15E-12	0.00	3.45E-05
5/5/46	*Zoobotryon verticillatum*→ *Phaeodactylum tricornutum*	5.46E-05	7.96E-10	200.00	200.00	24.00	0.13	8.94E+06	9.84E+07	8.24E-13	0.00	8.61E-05
6/47/45	*Mytilus edulis*→ *Rhodomonas* sp.	9.86E-01	1.25E-10	14000.00	580.88	15.00	0.21	2.27E+09	2.69E+10	9.21E-11	0.00	2.96E-09
7/48/45	*Ostrea edulis*→ *Pavlova lutheri*	4.39E-06	2.30E-11	0.00	NA	21.00	0.00	1.20E+10	2.48E+11	9.52E-18	0.00	1.77E-05
8/49/45	*Calliopius laeviusculus*→ *Mallotus villosus*	6.97E-03	8.39E-04	1000.00	100.00	14.50	0.33	1.00E+02	8.30E+03	1.63E-05	0.00	1.94E-02
8/49/45	*Calliopius laeviusculus*→ *Mallotus villosus*	6.58E-02	8.39E-04	1000.00	100.00	7.50	0.33	1.00E+02	7.20E+03	3.28E-05	0.00	3.38E-02
8/49/45	*Calliopius laeviusculus*→ *Mallotus villosus*	1.95E-01	8.39E-04	1000.00	100.00	7.50	1.00	1.00E+02	7.20E+03	9.32E-06	0.00	7.49E-02
9/50/48	*Callinectes sapidus*→ *Crassostrea virginica*	4.29E+02	3.49E+00	72000.00	3300.00	13.50	1.00	1.52E+01	1.52E+02	1.32E-02	1.00	2.26E-01
9/50/48	*Callinectes sapidus*→ *Crassostrea virginica*	4.29E+02	3.49E+00	72000.00	3300.00	19.50	1.00	1.52E+01	1.52E+02	6.24E-02	1.79	7.51E-02
9/50/48	*Callinectes sapidus*→ *Crassostrea virginica*	4.29E+02	3.49E+00	72000.00	3300.00	26.50	1.00	1.52E+01	1.52E+02	9.46E-02	1.00	3.29E-02
10/50/51	*Callinectes sapidus*→ *Macoma balthica*	4.29E+02	4.52E+00	NA	2341.40	26.00	2.00	4.27E+00	1.03E+02	3.38E-01	1.22	1.36E-01
10/50/51	*Callinectes sapidus*→ *Macoma balthica*	4.29E+02	4.52E+00	NA	2341.40	26.00	2.00	4.27E+00	1.03E+02	2.11E-01	1.24	1.66E-01
10/50/51	*Callinectes sapidus*→ *Macoma balthica*	5.21E+02	4.52E+00	NA	7420.32	26.00	2.00	2.70E+00	1.08E+01	8.46E-01	1.00	1.34E-01
11//11/11	*Mytilus edulis*→ *Isochrysis* sp.	1.49E+00	7.80E-11	5000.00	NA	14.00	0.04	3.24E+04	4.96E+05	5.83E-12	0.00	9.80E-06
11//11/11	*Mytilus edulis*→ *Phaeodactylum tricornutum*	1.49E+00	1.28E-08	5000.00	NA	14.00	0.04	3.00E+04	5.35E+05	1.02E-09	0.00	2.24E-05
11//11/11	*Cerastoderma edule*→ sediment	1.40E+00	2.81E-09	5000.00	NA	14.00	0.04	3.24E+04	4.30E+05	2.71E-10	0.00	2.19E-05
11//11/11	*Cerastoderma edule*→ sediment	1.40E+00	2.68E-10	5000.00	NA	14.00	0.04	2.94E+04	5.60E+05	1.44E-11	0.00	4.32E-06

11//11/11	*Cerastoderma edule*→ *Phaeodactylum tricornutum*	1.40E+00	1.28E-08	5000.00	NA	14.00	0.04	1.74E+04	4.82E+05	1.93E-09	0.00	1.83E-04
11//11/11	*Venerupis pullastra*→ *Phaeodactylum tricornutum*	1.60E+00	7.80E-11	5000.00	NA	14.00	0.04	3.18E+04	4.57E+05	4.54E-12	0.72	9.30E-05
11//11/11	*Venerupis pullastra*→ *Isochrysis sp.*	1.60E+00	1.28E-08	5000.00	NA	14.00	0.04	3.06E+04	4.78E+05	2.28E-09	0.00	2.34E-04
12//52/51	*Cancer magister*→ *Macoma balthica*	3.46E-01	1.05E-01	10000.00	1000.00	15.00	1.00	6.00E+01	1.20E+03	3.98E-03	0.00	9.84E-02
12//52/51	*Cancer magister*→ *Macoma balthica*	2.00E+00	1.05E-01	10000.00	1000.00	15.00	1.00	6.00E+01	1.20E+03	1.22E-03	0.53	4.78E-02
13/53/54	*Urosalpinx cinerea*→ *Balanus balanoides*	3.18E+00	9.03E-03	363.00	121.00	18.91	1.03	3.31E+02	5.29E+03	7.89E-06	1.00	2.95E-01
13/53/54	*Urosalpinx cinerea*→ *Balanus balanoides*	1.86E+00	9.03E-03	363.00	121.00	18.91	1.03	3.31E+02	5.29E+03	1.57E-05	1.00	3.61E-01
13/53/54	*Urosalpinx cinerea*→ *Balanus balanoides*	9.76E-01	9.03E-03	363.00	121.00	18.91	1.03	3.31E+02	5.29E+03	2.12E-05	0.00	1.39E-01
14/14/49	*Upogebia deltaura*→ *Artemia salina*	2.00E+00	3.51E-05	9100.00	NA	14.00	0.08	9.89E+02	1.41E+05	1.50E-07	0.74	8.93E-05
15/55/15	*Abarenicola pacifica*→ sediment organics	4.26E-01	1.00E-03	500.00	500.00	15.00	1.00	2.50E+04	6.24E+04	2.57E-07	0.00	0.00E+00
16/50/56	*Callinectes sapidus*→ *Mya arenaria*	3.12E+02	4.86E+00	90000.00	3600.00	25.00	3.00	5.56E+00	8.89E+01	1.22E-02	2.08	6.73E-01
16/50/56	*Callinectes sapidus*→ *Mya arenaria*	3.12E+02	4.86E+00	90000.00	3600.00	25.00	3.00	5.56E+00	8.89E+01	6.65E-01	1.00	5.04E-01
17/53/47	*Acanthina spirata*→ *Mytilus edulis*	4.48E+00	4.70E-01	924.00	132.00	14.00	21.00	7.58E+02	3.79E+03	1.39E-04	0.00	4.60E+00
17/53/47	*Thais emarginata*→ *Mytilus edulis*	4.48E+00	4.70E-01	924.00	132.00	14.00	21.00	3.79E+02	3.41E+03	7.43E-05	0.00	3.46E+00
18/57/45	*Ciona intestinalis*→ *Rhodomonas* sp.	2.18E-01	1.13E-10	1000.00	NA	15.40	0.04	1.21E+09	1.90E+10	2.23E-12	0.00	9.17E-18
18/57/45	*Ciona intestinalis*→ *Rhodomonas* sp.	2.74E+00	1.13E-10	1000.00	NA	15.40	0.04	1.68E+09	2.56E+10	8.21E-13	0.00	6.92E-09
19/57/45	*Ciona intestinalis*→ *Rhodomonas* sp.	1.90E+00	1.13E-10	5000.00	314.16	10.00	0.04	9.44E+08	7.57E+09	1.37E-11	0.00	4.46E-09
19/57/45	*Ciona intestinalis*→ *Rhodomonas* sp.	7.81E-01	1.13E-10	5000.00	314.16	10.00	0.04	5.03E+08	8.21E+09	2.71E-11	0.00	1.33E-08
19/57/45	*Ciona intestinalis*→ sediment	2.80E+00	1.09E-08	3000.00	NA	15.00	0.04	1.13E+04	8.47E+04	4.60E-11	1.00	7.23E-04

20/57/20	*Ascidiella scabra*→ sediment	3.55E+00	1.09E-08	3000.00	NA	15.00	0.04	8.33E+03	1.74E+05	3.79E-10	0.00	1.91E-03
20/57/20	*Ascidiella scabra*→ sediment	5.18E-01	1.09E-08	3000.00	NA	15.00	0.04	5.00E+03	1.09E+05	9.65E-12	1.00	6.55E-03
20/57/20	*Ciona intestinalis*→ sediment	3.05E+00	4.77E-08	3000.00	NA	15.00	0.04	1.67E+03	4.53E+04	2.32E-08	0.00	4.09E-03
20/57/20	*Ascidiella scabra*→ sediment	3.77E+00	4.77E-08	3000.00	NA	15.00	0.04	5.00E+03	5.70E+04	1.98E-09	0.00	7.27E-03
20/57/20	*Ascidiella scabra*→ sediment	5.18E-01	4.77E-08	3000.00	NA	15.00	0.04	2.00E+03	4.40E+04	1.04E-09	0.00	2.22E-02
20/57/20	*Ciona intestinalis*→ sediment	3.05E+00	1.80E-08	3000.00	NA	15.00	0.04	1.67E+03	3.73E+04	3.63E-10	1.00	2.98E-03
20/57/20	*Ascidiella scabra*→ sediment	3.77E+00	1.80E-08	3000.00	NA	15.00	0.04	2.00E+03	4.30E+04	5.05E-10	0.00	3.41E-03
20/57/20	*Ascidiella scabra*→ sediment	4.74E-01	1.80E-08	3000.00	NA	15.00	0.04	2.33E+03	3.30E+04	6.66E-10	0.00	1.95E-02
20/57/20	*Ciona intestinalis*→ sediment	9.52E-02	1.09E-08	3000.00	NA	15.00	0.04	6.67E+03	1.06E+05	4.92E-11	1.00	1.23E-03
20/57/20	*Ciona intestinalis*→ sediment	1.09E+00	1.09E-08	3000.00	NA	15.00	0.04	4.00E+03	1.29E+05	1.94E-10	0.00	4.98E-03
20/57/20	*Ciona intestinalis*→ sediment	5.66E+00	1.09E-08	3000.00	NA	15.00	0.04	7.33E+03	1.46E+05	1.00E-10	0.00	8.60E-03
20/57/20	*Ciona intestinalis*→ sediment	1.82E+00	1.09E-08	3000.00	NA	5.00	0.04	7.33E+03	1.04E+05	2.86E-11	1.00	4.81E-03
20/57/20	*Ciona intestinalis*→ sediment	1.82E+00	1.09E-08	3000.00	NA	10.00	0.04	3.67E+03	1.16E+05	7.99E-12	1.00	4.40E-03
20/57/20	*Ciona intestinalis*→ sediment	1.82E+00	1.09E-08	3000.00	NA	15.00	0.04	4.33E+03	1.09E+05	8.35E-11	0.00	2.06E-03
21/58/59	*Littorina littorea*→ periphyton (unspecified)	1.74E+00	1.00E-04	250.00	56.75	14.00	1.00	5.46E+03	4.97E+05	1.75E-06	0.00	3.81E-03
22/60/22	*Abarenicola pacifica*→ *sediment organics*	9.43E-02	1.00E+00	729.00	83.00	12.00	10.00	2.77E+03	8.19E+03	2.13E-04	0.00	6.52E-01
22/60/22	*Abarenicola pacifica*→ *sediment organics*	9.43E-02	1.00E+00	729.00	83.00	12.00	10.00	1.33E+03	2.80E+04	2.36E-06	1.00	1.27E+00
22/60/22	*Abarenicola pacifica*→ *sediment organics*	9.43E-02	1.00E+00	729.00	83.00	12.00	10.00	4.82E+02	2.98E+04	2.94E-06	1.00	2.37E+00
23/23/23	*Haliotis asinina*→ macroalgae (unspecified)	8.24E+00	8.24E-02	75600.00	2160.00	27.00	1.00	1.10E+02	4.37E+02	3.85E-02	0.00	3.65E-04

24/43/61	*Crangon septemspinosa→ Pseudopleuronectes americanus*	1.21E+00	3.53E-02	69400.00	2231.23	10.00	1.00	4.48E+00	8.96E+01	1.46E-04	2.61	2.75E-01
24/43/61	*Crangon septemspinosa→ Pseudopleuronectes americanus*	1.21E+00	3.53E-02	69400.00	2231.23	16.00	1.00	4.48E+00	8.96E+01	1.68E-02	0.00	2.24E-01
25/62/47	*Panopeus herbstii→ Brachidontes exustus*	3.11E+01	3.20E+01	42000.00	1400.00	30.15	1.00	1.43E+01	2.57E+02	5.49E+00	0.00	1.75E-01
25/62/47	*Panopeus herbstii→ Brachidontes exustus*	3.20E+01	2.05E-01	42000.00	1400.00	30.22	1.00	1.43E+01	2.57E+02	1.37E-02	1.00	1.55E-01
25/62/47	*Panopeus herbstii→ Brachidontes exustus*	5.90E+01	2.05E-01	42000.00	1400.00	28.10	1.00	1.43E+01	2.57E+02	4.01E-02	0.00	1.02E-01
25/62/47	*Panopeus herbstii→ Brachidontes exustus*	5.67E+01	2.05E-01	42000.00	1400.00	27.45	1.00	1.43E+01	2.57E+02	6.51E-02	0.00	1.12E-01
26/63/64	*Fundulus heteroclitus→ Orchestia grillus*	6.22E-01	3.00E-03	60000.00	10000.00	21.00	0.08	8.00E+00	6.40E+01	1.17E-03	1.00	2.49E-03
26/63/64	*Fundulus heteroclitus→ Orchestia grillus*	6.22E-01	3.00E-02	60000.00	10000.00	21.00	0.08	8.00E+00	6.40E+01	2.30E-02	1.00	6.55E-03
26/63/64	*Fundulus heteroclitus→ Orchestia grillus*	6.22E-01	3.00E-03	60000.00	10000.00	21.00	0.08	4.00E+00	3.20E+01	7.29E-03	1.00	2.63E-03
26/63/64	*Fundulus heteroclitus→ Orchestia grillus*	6.22E-01	3.00E-02	60000.00	10000.00	21.00	0.08	4.00E+00	3.20E+01	1.40E-02	1.00	7.13E-03
27/43/65	*Crangon crangon→ Pleuronectes platessa*	2.64E-01	2.18E-02	370000.00	10000.00	14.10	0.50	2.00E+00	6.40E+01	1.47E-03	1.43	3.67E-02
28/28/45	*Mytilus edulis→* seston (unspecified)	4.67E-01	4.71E-10	500.00	NA	12.00	0.08	1.40E+04	5.30E+05	5.23E-12	0.21	1.25E-03
28/28/45	*Mytilus edulis→* seston (unspecified)	1.64E+00	4.71E-10	500.00	NA	12.00	0.08	1.60E+04	6.88E+05	4.64E-12	0.87	8.72E-04
28/28/45	*Mytilus edulis→* seston (unspecified)	3.50E+00	4.71E-10	500.00	NA	12.00	0.08	8.00E+03	6.62E+05	3.87E-12	0.52	5.31E-04
29/48/45	*Ostrea edulis→ Isochrysis galbana*	3.32E-06	2.76E-11	1.00	NA	26.00	0.08	4.75E+10	4.14E+11	3.31E-15	0.00	5.60E-06
29/48/45	*Ostrea edulis→ Dunaliella tertiolecta*	3.32E-06	8.71E-11	1.00	NA	26.00	0.08	2.51E+10	1.20E+11	1.05E-14	0.00	1.09E-05
29/48/45	*Ostrea edulis→ Isochrysis galbana*	2.62E-04	2.76E-11	1.00	NA	26.00	0.08	6.20E+10	4.06E+11	1.36E-16	0.00	0.00E+00
29/48/45	*Ostrea edulis→ Dunaliella tertiolecta*	2.62E-04	8.71E-11	1.00	NA	26.00	0.08	2.25E+10	2.16E+11	1.05E-14	0.00	1.64E-06

30/52/66	*Cancer irroratus→ Placopecten magellanicus*	1.93E+02	5.59E+00	282743.20	11309.73	12.70	1.00	1.77E+00	4.42E+01	5.40E+00	0.00	7.95E-02
31/67/66	*Asterias vulgaris→ Placopecten magellanicus*	3.44E+01	8.13E-01	NA	1800.00	11.23	1.00	5.56E+00	6.22E+02	1.49E-03	1.00	1.63E-01
31/67/66	*Asterias vulgaris→ Placopecten magellanicus*	3.29E+01	9.32E-01	NA	1800.00	11.35	1.00	6.11E+01	6.17E+02	1.77E-03	1.00	1.56E-01
32/52/66	*Cancer irroratus→ Placopecten magellanicus*	2.08E+02	2.72E+00	NA	10000.00	11.20	1.00	1.10E+01	1.11E+02	7.04E-01	0.00	3.20E-01
32/52/66	*Cancer irroratus→ Placopecten magellanicus*	2.08E+02	2.72E+00	NA	10000.00	13.50	1.00	1.00E+01	1.11E+02	7.20E-01	0.00	3.61E-01
33/68/69	*Enchinogammarus marinus→ Jaera nordmanni*	3.60E-02	5.70E-04	250.00	44.18	12.00	0.50	4.53E+02	4.53E+03	1.62E-05	0.00	4.96E-02
33/68/69	*Enchinogammarus marinus→ Jaera nordmanni*	3.60E-02	5.70E-04	250.00	44.18	12.00	0.50	4.53E+02	4.53E+03	7.80E-06	0.00	3.15E-02
33/68/69	*Enchinogammarus marinus→ Jaera nordmanni*	3.60E-02	5.70E-04	250.00	44.18	12.00	1.67	4.53E+02	9.05E+03	3.89E-06	0.00	1.23E-01
33/68/69	*Enchinogammarus marinus→ Jaera nordmanni*	3.60E-02	5.70E-04	250.00	44.18	12.00	1.67	4.53E+02	9.05E+03	1.67E-07	0.70	1.29E-01
34/68/69	*Enchinogammarus marinus→ Jaera nordmanni*	1.78E-03	3.11E-05	2.69	2.99	12.00	1.00	6.70E+03	1.34E+05	5.35E-09	0.00	3.07E-01
34/68/69	*Enchinogammarus marinus→ Jaera nordmanni*	1.97E-02	5.80E-04	72.57	26.88	12.00	1.00	7.44E+02	1.49E+04	2.98E-06	0.00	1.63E-01
34/68/69	*Enchinogammarus marinus→ Jaera nordmanni*	6.03E-02	2.26E-03	335.98	74.66	12.00	1.00	2.68E+02	5.36E+03	1.17E-05	0.00	1.09E-01
34/68/69	*Enchinogammarus marinus→ Jaera nordmanni*	6.03E-02	3.11E-05	335.98	74.66	12.00	1.00	2.68E+02	5.36E+03	8.59E-09	0.00	5.03E-01
34/68/69	*Enchinogammarus marinus→ Jaera nordmanni*	6.03E-02	3.11E-05	335.98	74.66	12.00	1.00	2.68E+02	5.36E+03	2.85E-07	0.00	5.55E-02
34/68/69	*Enchinogammarus marinus→ Jaera nordmanni*	3.37E-02	6.46E-04	250.00	44.18	12.00	1.00	4.53E+02	9.05E+03	4.64E-08	1.00	8.89E-01
34/68/69	*Enchinogammarus marinus→ Jaera nordmanni*	3.37E-02	6.46E-04	250.00	44.18	12.00	1.00	4.53E+02	9.05E+03	7.73E-08	1.00	3.91E-01
34/68/69	*Enchinogammarus marinus→ Jaera nordmanni*	3.37E-02	6.46E-04	250.00	44.18	12.00	1.00	4.53E+02	9.05E+03	1.47E-06	1.00	1.29E-01
34/68/69	*Enchinogammarus marinus→ Jaera nordmanni*	3.37E-02	6.46E-04	250.00	44.18	12.00	1.00	4.53E+02	9.05E+03	1.13E-05	0.00	1.72E-01
35/53/47	*Trochia cingulata→ Mytilus galloprovincialis*	1.74E+00	4.70E-01	500.00	78.54	15.00	40.00	1.27E+02	7.64E+02	8.51E-05	0.00	4.16E+01

35/53/47	*Trochia cingulata→ Semimytilus algosus*	1.74E+00	4.70E-01	500.00	78.54	14.00	40.00	1.27E+02	7.64E+02	6.06E-05	0.00	1.87E+00
35/53/47	*Trochia cingulata→ Aulacomya atra*	1.74E+00	4.70E-01	500.00	78.54	14.00	40.00	1.27E+02	7.64E+02	4.70E-05	0.00	0.00E+00
36/36/36	*Hemimysis anomala→ Chelicorophium curvispinum*	1.75E-02	3.90E-03	700.00	103.87	12.00	1.00	2.86E+03	1.43E+05	2.18E-07	0.00	7.73E-02
36/36/36	*Hemimysis anomala→ Chelicorophium curvispinum*	1.75E-02	3.90E-03	700.00	103.87	12.00	1.00	1.93E+02	9.63E+03	4.22E-06	0.12	1.74E-01
36/36/36	*Hemimysis anomala→ Chelicorophium curvispinum*	1.75E-02	3.90E-03	700.00	103.87	12.00	1.00	1.93E+02	9.63E+03	2.92E-06	0.26	4.11E-01
36/36/36	*Gammarus d. celticus→ Chelicorophium curvispinum*	8.13E-03	3.90E-03	700.00	103.87	12.00	1.00	2.86E+03	1.43E+05	5.54E-09	1.00	3.60E-01
36/36/36	*Gammarus d. celticus→ Chelicorophium curvispinum*	2.24E-02	3.90E-03	700.00	103.87	12.00	1.00	2.86E+03	1.43E+05	8.67E-07	0.36	2.23E-01
36/36/36	*Gammarus d. celticus→ Chelicorophium curvispinum*	2.24E-02	3.90E-03	700.00	103.87	12.00	1.00	1.93E+02	9.63E+03	4.49E-06	0.47	8.09E-01
36/36/36	*Gammarus d. celticus→ Chelicorophium curvispinum*	2.24E-02	3.90E-03	700.00	103.87	12.00	1.00	1.93E+02	9.63E+03	1.89E-05	0.00	5.04E-06
36/36/36	*Gammarus d. celticus→ Chelicorophium curvispinum*	5.38E-02	3.90E-03	700.00	103.87	12.00	1.00	2.86E+03	1.43E+05	4.92E-06	0.01	1.24E-01
36/36/36	*Gammarus d. celticus→ Chelicorophium curvispinum*	5.38E-02	3.90E-03	700.00	103.87	12.00	1.00	1.93E+02	9.63E+03	2.00E-06	0.21	1.03E-01
36/36/36	*Gammarus d. celticus→ Chelicorophium curvispinum*	5.38E-02	3.90E-03	700.00	103.87	12.00	1.00	1.93E+02	9.63E+03	1.73E-05	0.08	1.66E-01
36/36/36	*Gammarus d. celticus→ Chelicorophium curvispinum*	8.04E-02	3.90E-03	700.00	103.87	12.00	1.00	2.86E+03	1.43E+05	1.02E-07	0.04	3.79E-02
36/36/36	*Gammarus d. celticus→ Chelicorophium curvispinum*	8.04E-02	3.90E-03	700.00	103.87	12.00	1.00	1.93E+02	9.63E+03	2.16E-06	0.06	5.78E-02
36/36/36	*Gammarus d. celticus→ Chelicorophium curvispinum*	8.04E-02	3.90E-03	700.00	103.87	12.00	1.00	1.93E+02	9.63E+03	7.68E-06	0.44	2.11E-01
36/36/36	*Pungitius pungitius→ Chelicorophium curvispinum*	1.57E-01	3.90E-03	700.00	103.87	12.00	1.00	2.86E+03	1.43E+05	8.04E-06	0.00	3.35E-02
36/36/36	*Pungitius pungitius→ Chelicorophium curvispinum*	1.57E-01	3.90E-03	700.00	103.87	12.00	1.00	1.93E+02	9.63E+03	7.00E-05	0.00	3.41E-02
36/36/36	*Pungitius pungitius→ Chelicorophium curvispinum*	1.57E-01	3.90E-03	700.00	103.87	12.00	1.00	1.93E+02	9.63E+03	6.79E-06	0.00	6.20E-07
36/36/36	*Gasterosteus aculeatus→ Chelicorophium curvispinum*	4.03E-01	3.90E-03	700.00	103.87	12.00	1.00	2.86E+03	1.43E+05	1.13E-05	0.00	2.96E-02

36/36/36	*Gasterosteus aculeatus→ Chelicorophium curvispinum*	4.03E-01	3.90E-03	700.00	103.87	12.00	1.00	1.93E+02	9.63E+03	1.06E-06	0.00	2.76E-02
36/36/36	*Gasterosteus aculeatus→ Chelicorophium curvispinum*	4.03E-01	3.90E-03	700.00	103.87	12.00	1.00	1.93E+02	2.89E+04	1.12E-05	0.11	1.81E-02
36/36/36	*Gasterosteus aculeatus→ Chelicorophium curvispinum*	6.65E-01	3.90E-03	700.00	103.87	12.00	1.00	2.86E+03	1.43E+05	8.27E-06	0.00	1.27E-02
36/36/36	*Gasterosteus aculeatus→ Chelicorophium curvispinum*	6.65E-01	3.90E-03	700.00	103.87	12.00	1.00	1.93E+02	9.63E+03	7.34E-05	0.07	1.54E-02
36/36/36	*Gasterosteus aculeatus→ Chelicorophium curvispinum*	6.65E-01	3.90E-03	700.00	103.87	12.00	1.00	1.93E+02	2.89E+04	6.57E-06	0.26	3.85E-02
36/36/36	*Gasterosteus aculeatus→ Chelicorophium curvispinum*	1.23E+00	3.90E-03	700.00	103.87	12.00	1.00	2.86E+03	1.43E+05	8.55E-06	0.00	6.74E-03
36/36/36	*Gasterosteus aculeatus→ Chelicorophium curvispinum*	1.23E+00	3.90E-03	700.00	103.87	12.00	1.00	1.93E+02	9.63E+03	3.52E-05	0.13	1.01E-02
36/36/36	*Gasterosteus aculeatus→ Chelicorophium curvispinum*	1.23E+00	3.90E-03	700.00	103.87	12.00	1.00	1.93E+02	2.89E+04	9.06E-06	0.12	6.88E-03
36/36/36	*Salmo trutta→ Chelicorophium curvispinum*	1.87E+00	3.90E-03	700.00	103.87	12.00	1.00	2.86E+03	1.43E+05	1.05E-06	0.23	3.17E-02
36/36/36	*Salmo trutta→ Chelicorophium curvispinum*	1.87E+00	3.90E-03	700.00	103.87	12.00	1.00	1.93E+02	9.63E+03	7.29E-06	0.26	1.79E-02
36/36/36	*Salmo trutta→ Chelicorophium curvispinum*	1.87E+00	3.90E-03	700.00	103.87	12.00	1.00	1.93E+02	9.63E+03	5.76E-06	0.02	4.50E-08
36/36/36	*Salmo trutta→ Chelicorophium curvispinum*	6.43E+00	3.90E-03	700.00	103.87	12.00	1.00	2.86E+03	5.71E+04	1.21E-07	0.62	5.43E-02
36/36/36	*Salmo trutta→ Chelicorophium curvispinum*	6.43E+00	3.90E-03	700.00	103.87	12.00	1.00	1.93E+02	3.85E+03	1.61E-06	0.70	2.98E-02
36/36/36	*Salmo trutta→ Chelicorophium curvispinum*	6.43E+00	3.90E-03	700.00	103.87	12.00	1.00	1.93E+02	3.85E+03	2.80E-06	0.00	2.64E-02
37/37/37	*Gammarus d. celticus→ Chelicorophium curvispinum*	9.42E-02	5.40E-03	500.00	153.94	12.00	1.00	6.50E+01	2.60E+03	2.55E-05	0.20	1.38E-01
37/37/37	*Gammarus d. celticus→ Chelicorophium curvispinum*	9.42E-02	5.40E-03	500.00	153.94	12.00	1.00	6.50E+01	2.60E+03	3.94E-05	0.09	7.55E-02
37/37/37	*Gammarus d. celticus→ Chelicorophium curvispinum*	9.42E-02	5.40E-03	500.00	153.94	12.00	1.00	6.50E+01	2.60E+03	7.39E-05	0.05	6.53E-02
38/70/68	*Scyliorhinus canicula→ Echinogammarus marinus*	1.42E+02	1.32E-02	60000.00	1970.25	11.30	0.08	5.08E+00	1.02E+02	2.67E-02	0.00	8.82E-03
38/70/68	*Scyliorhinus canicula→ Echinogammarus marinus*	1.42E+02	1.32E-02	60000.00	1970.25	16.30	0.08	5.08E+00	1.02E+02	3.17E-03	1.00	9.97E-03

38/70/68	*Scyliorhinus canicula→ Echinogammarus marinus*	1.42E+02	1.32E-02	60000.00	1970.25	16.30	0.08	5.08E+00	1.02E+02	1.57E-02	0.00	0.00E+00
38/70/68	*Scyliorhinus canicula→ Echinogammarus marinus*	1.42E+02	1.32E-02	60000.00	1970.25	16.30	0.08	5.08E+00	1.02E+02	1.16E-02	0.73	7.32E-03
38/70/68	*Scyliorhinus canicula→ Echinogammarus marinus*	1.42E+02	1.32E-02	60000.00	1970.25	16.30	0.08	5.08E+00	1.02E+02	1.69E-02	0.00	1.03E-02

Table S2 Consumption data sources. Source numbers are referenced in the first column of Table S1.

	**data source (supplementary experiments or published studies)**
1	Supplementary experiments (refer to section 2 for methods and details).
2	Aljetlawi, A.A., Sparrevik, E. & Leonardsson, K. (2004) Prey-predator size-dependent functional response: derivation and rescaling to the real world. Journal of Animal Ecology, 73, 239–252.
3	Anderson, T.W. (2001) Predator responses, prey refuges, and density-dependent mortality of a marine fish. Ecology, 82, 245–257.
4	Andresen, H. & van der Meer, J. (2010) Brown shrimp (*Crangon crangon*, L.) functional response to density of different sized juvenile bivalves *Macoma balthica* (L.). Journal of Experimental Marine Biology and Ecology, 390, 31–38.
5	Bullivant, J.S. (1968) The rate of feeding of the bryozoan, *Zoobotryon verticillatum*. New Zealand Journal of Marine and Freshwater Research, 2, 111–134.
6	Clausen, I. & Riisgaard, H.U. (1996) Growth, filtration and respiration in the mussel *Mytilus edulis*: no evidence for physiological regulation of the filter-pump to nutritional needs. Marine Ecology Progress Series, 141, 37–45.
7	Crisp, D.J., Yule, A.B. & White, K.N. (1985) Feeding by oyster larvae: the functional response, energy budget and a comparison with mussel larvae. Journal of the Marine Biological Association of the United Kingdom, 65, 759–783.
8	Deblois, E.M. & Leggett, W.C. (1991) Functional response and potential impact of invertebrate predators on benthic fish eggs: analysis of the *Calliopius laeviusculus*-capelin (*Mallotus villosus*) predator-prey system*. Marine Ecology Progress Series, 69, 205–216.
9	Eggleston, D.B. (1990) Behavioural mechanisms underlying variable functional responses of blue crabs, *Callinectes sapidus* feeding on juvenile oysters, *Crassostrea virginica*. Journal of Animal Ecology, 59, 615–630.
10	Eggleston, D.B., Lipcius, R.N. & Hines, A.H. (1992) Density-dependent predation by blue crabs upon infaunal clam species with contrasting distribution and abundance patterns. Marine Ecology Progress Series, 85, 55–68.
11	Foster-Smith, R.L. (1975) The effect of concentration of suspension on the filtration rates and pseudofaecal production for *Mytilus edulis* L., *Cerastoderma edule* (L.) and *Venerupis pullastra* (Montagu). Journal of Experimental Marine Biology and Ecology, 17, 1–22.
12	Iribarne, O., Armstrong, D. & Fernbndez, M. (1995) Environmental impact of intertidal juvenile dungeness crab habitat enhancement: effects on bivalves and crab foraging rate. Journal of Experimental Marine Biology and Ecology, 192, 173–194.
13	Katz, C.H. (1985) A nonequilibrium marine predator-prey interaction. Ecology, 66, 1426–1438.
14	Lindahl, U. & Baden, S.P. (1997) Type three functional response in filter feeding of the burrowing shrimp *Upogebia deltaura* (Leach). Ophelia, 47, 33–41.

15	Linton, D.L. & Taghon, G.L. (2000) Feeding, growth, and fecundity of *Abarenicola pacifica* in relation to sediment organic concentration. Marine Ecology Progress Series, 205: 229-240. Journal of Experimental Marine Biology and Ecology, 254, 85–107.
16	Lipcius, R.N. & Hines, A.H. (1986) Variable functional responses of a marine predator in dissimilar homogeneous microhabitats. Ecology, 67, 1361–1371.
17	Murdoch, W.W. (1969) Switching in general predators: experiments on predator specificity and stability of prey populations. Ecological Monographs, 39, 335–354.
18	Petersen, J.K. & Riisgard, H.U. (1992) Filtration capacity of the ascidian *Ciona intestinalis* and its grazing impact in a shallow fjord. Marine Ecology Progress Series, 88, 9–17.
19	Petersen, J.K., Schou, O. & Thor, P. (1995) Growth and energetics in ascidian *Ciona intestinalis*. Marine Ecology Progress Series, 120, 175–184.
20	Robbins, I.J. (1983) The effects of body size, temperature, and suspension density on the filtration and ingestion of inorganic particulate suspensions by ascidians. Journal of Experimental Marine Biology and Ecology, 70, 65–78.
21	Sommer, U. (1999) The susceptibility of benthic microalgae to periwinkle (*Littorina littorea*, Gastropoda) grazing in laboratory experiments. Aquatic Botany, 63, 11–21.
22	Taghon, G.L. & Greene, R.R. (1990) Effects of sediment-protein concentration on feeding and growth rates of *Abarenicola pacifica* Healy et Wells (Polychaeta: Arenicolidae). Journal of Experimental Marine Biology and Ecology, 136, 197–216.
23	Tahil, A.S. & Juinio-Menez, M.A. (1999) Natural diet, feeding periodicity and functional response to food density of the abalone, *Haliotis asinina* L., (Gastropoda). Aquaculture Research, 30, 95–107.
24	Taylor, D.L. & Collie, J.S. (2003) Effect of temperature on the functional response and foraging behaviour of the sand shrimp *Crangon septemspinosa* preying on juvenile winter flounder *Pseudopleuronectes americanus*. Marine Ecology Progress Series, 263, 217–234.
25	Toscano, B.J. & Griffen, B.D. (2014) Trait-mediated functional responses: predator behavioural type mediates prey consumption. Journal of Animal Ecology, 83, 1469–1477.
26	Vince, S., Valiela, I., Backus, N. & Teal, J.M. (1976) Predation by the salt marsh killifish *Fundulus heteroclitus* (L.) in relation to prey size and habitat structure: Consequences for prey distribution and abundance. Journal of Experimental Marine Biology and Ecology, 23, 255–266.
27	Wennhage, H. (2002) Vulnerability of newly settled plaice (*Pleuronectes platessa* L.) to predation: effects of habitat structure and predator functional response. Journal of Experimental Marine Biology and Ecology, 269, 129–145.
28	Widdows, J., Fieth, P. & Worrall, C.M. (1979) Relationships between seston, available food and feeding activity in the common mussel *Mytilus edulis*. Marine Biology, 50, 195–207.
29	Wilson, J.H. (1980) Particle retention and selection by larvae and spat of *Ostrea edulis* in algal suspensions. Marine Biology, 57, 135–145.

30	Wong, M.C. & Barbeau, M.A. (2005) Prey selection and the functional response of sea stars (*Asterias vulgaris* Verrill) and rock crabs (*Cancer irroratus* Say) preying on juvenile sea scallops (*Placopecten magellanicus* (Gmelin)) and blue mussels (*Mytilus edulis* Linnaeus). Journal of Experimental Marine Biology and Ecology, 327, 1–21.
31	Wong, M.C., Barbeau, M.A., Dowd, M. & Richard, K.R. (2006) Behavioural mechanisms underlying functional response of sea stars *Asterias vulgaris* preying on juvenile sea scallops *Placopecten magellanicus*. Marine Ecology Progress Series, 317, 75–86.
32	Wong, M.C. & Barbeau, M.A. (2006) Rock crab predation of juvenile sea scallops: the functional response and its implications for bottom culture. Aquaculture International, 14, 355–376.
33	Alexander, M.E., Dick, J.T.A., O'Connor, N.E., Haddaway, N.R. & Farnsworth, K.D. (2012) Functional responses of the intertidal amphipod *Echinogammarus marinus*: effects of prey supply, model selection and habitat complexity. Marine Ecology Progress Series, 468, 191–202.
34	Alexander, M.E., Dick, J.T.A. & O'Connor, N.E. (2013) Born to kill: predatory functional responses of the littoral amphipod *Echinogammarus marinus* Leach throughout its life history. Journal of Experimental Marine Biology and Ecology, 439, 92–99.
35	Alexander, M.E., Raven, H.J. & Robinson, T.B. (2015) Foraging decisions of a native whelk, *Trochia cingulata* Linnaeus, and the effects of invasive mussels on prey choice. Journal of Experimental Marine Biology and Ecology, 470, 26–33.
36	Barrios-O'Neill, D., Kelly, R., Dick, J.T.A., Ricciardi, A., MacIsaac, H.J. & Emmerson, M.C. (2016) On the context-dependent scaling of consumer feeding rates. Ecology Letters, 19, 668–678.
37	Barrios-O'Neill, D., Dick, J.T.A., Emmerson, M.C., Ricciardi, A. & MacIsaac, H.J. (2015) Predator-free space, functional responses and biological invasions. Functional Ecology, 29, 377–384.
38	South, J. & Dick, J.T.A. (2017) Effects of acute and chronic temperature changes on the functional responses of the dogfish *Scyliorhinus canicula* (Linnaeus, 1758) towards amphipod prey *Echinogammarus marinus* (Leach, 1815). Environmental Biology of Fishes, 100, 1251-1263.

Table S3 Mass conversion factor data sources. In the conversion column, any formulae with log transformed predictors give log transformed mass (either $\log_{10}$ or $\log_e$ as specified in each equation). Source numbers are referenced in the first column of Table S1.

source	conversion type	Source	conversion
39	*dry > wet*	Ricciardi A, Bourget E (1998) Weight-to-weight conversion factors for marine benthic macroinvertebrates. Mar Ecol Prog Ser 163:245–251.	(dry/20.7) * 100
40	*dry > wet*	Ricciardi A, Bourget E (1998) Weight-to-weight conversion factors for marine benthic macroinvertebrates. Mar Ecol Prog Ser 163:245–251.	(dry/20) * 100
41	*length > wet*	Froese R, Thorson JT, Reyes RB (2014) A Bayesian approach for estimating length-weight relationships in fishes. J Appl Ichthyol 30(1):78–85.	$0.00813 * \text{length}^{3.03}$
42	*length > wet*	Froese R, Thorson JT, Reyes RB (2014) A Bayesian approach for estimating length-weight relationships in fishes. J Appl Ichthyol 30(1):78–85.	$0.01318 * \text{length}^{3.05}$
43	*length > wet*	Haefner PJ (1973) Length-weight relationship of the sand shrimp, *Crangon septemspinosa*. Chesap Sci 14(2):141–143.	$-5.771 + 3.363 * \log_{10}(\text{length})$
44	*length > wet*	Rumohr H, Brey T, Ankar S (1987) A compilation of biometric conversion factors for benthic invertebrates of the Baltic Sea. Balt Mar Biol 9:1–56.	$-0.964 + 3.127 * \log_{10}(\text{length})$
45	*ESD > biovolume*	Sun J, Liu D (2003) Geometric models for calculating cell biovolume and surface area for phytoplankton. J Plankton Res 25(11):1331–1346.	$(\pi /6) * \text{ESD}^3$
46	*length > biovolume*	Sun J, Liu D (2003) Geometric models for calculating cell biovolume and surface area for phytoplankton. J Plankton Res 25(11):1331–1346.	$(\pi /4) * \text{length} * (\text{length}/4) * (\text{length}/4)$
47	*length > wet*	Rumohr H, Brey T, Ankar S (1987) A compilation of biometric conversion factors for benthic invertebrates of the Baltic Sea. Balt Mar Biol 9:1–56.	$-0.354 + 2.326 * \log_{10}(\text{length})$
48	*length > wet*	Chatterji A, Ansari A, Ingole B, Parulekar A (1985) Length-weight relationship of giant oyster, *Crassostrea gryphoides* (Schlotheim). Mahasagar - Bull Natl Inst Oceanogr 18(4):521–524.	$0.262*10^{-3} * \text{length}^{2.95}$
49	*length > wet*	Rumohr H, Brey T, Ankar S (1987) A compilation of biometric conversion factors for benthic invertebrates of the Baltic Sea. Balt Mar Biol 9:1–56.	$-0.795 + 2.19 * \log_{10}(\text{length})$
50	*length > wet*	de Araújo, M. & de Lira, J. (2013) Condition factor and carapace width versus wet weight relationship in the swimming crab *Callinectes danae* Smith 1869 (Decapoda: Portunidae) at the Santa Cruz Channel, Pernambuco State, Brazil. Nauplius 20(1):41–50.	$-4.1 + 3.12 * \log_e(\text{length})$
51	*length > wet*	Rumohr H, Brey T, Ankar S (1987) A compilation of biometric conversion factors for benthic invertebrates of the Baltic Sea. Balt Mar Biol 9:1–56.	$-0.964 + 3.127 * \log_{10}(\text{length})$

52	*length > wet*	Haefner PA, van Engel WA (2006) Aspects of molting, growth and survival of male rock crabs, *Cancer irroratus*, in Chesapeake Bay. Chesapeake Science 16(4): 253–265.	$-4.06 + 3.14 * \log_{10}(\text{length})$
53	*length > wet*	Aydın M, Düzgüneş E, Karadurmuş U (2016) Rapa whelk (*Rapana venosa* Valenciennes, 1846) fishery along the Turkish coast of the Black Sea. J Aquac Eng Fish Res 2(2):85–96.	$0.223 * \text{length}^{2.695}$
54	*length > wet*	Spivey HR (1989) The size variable and allometric analysis in the barnacle genus *Balanus*. J Nat Hist 23(5):1017–1032.	$-0.734 + 3.106 * \log_{10}(\text{length})$
55	*dry > wet*	Ricciardi A, Bourget E (1998) Weight-to-weight conversion factors for marine benthic macroinvertebrates. Mar Ecol Prog Ser 163:245–251.	(dry/17.6) * 100
56	*length > wet*	Rumohr H, Brey T, Ankar S (1987) A compilation of biometric conversion factors for benthic invertebrates of the Baltic Sea. Balt Mar Biol 9:1–56.	$-1.498 + 2.993 * \log_{10}(\text{length})$
57	*dry > wet*	Ricciardi A, Bourget E (1998) Weight-to-weight conversion factors for marine benthic macroinvertebrates. Mar Ecol Prog Ser 163:245–251.	(dry/6.2) * 100
58	*length > wet*	Rumohr, H., Brey, T. & Ankar, S. (1987) A compilation of biometric conversion factors for benthic invertebrates of the Baltic Sea. Baltic Marine Biology, 9, 1–56.	$-2.518 + 3.609 * \log_{10}(\text{length})$
59	*chlorophyll > wet*	Álvarez E., Nogueira E. & López-Urrutia. Á (2017) In vivo single-cell fluorescence and size scaling of phytoplankton chlorophyll content. Appl Environ Microbiol 83(7):1–16.	$\log_{10}(3)$ pg chl / $\log_{10}(5)$ μm^3 cell
60	*dry > wet*	Ricciardi A, Bourget E (1998) Weight-to-weight conversion factors for marine benthic macroinvertebrates. Mar Ecol Prog Ser 163:245–251.	(dry/17.6) * 100
61	*length > wet*	Kwak SN, Park JM (2016) Length–weight and length–length relationships for six flounder species (Pleuronectiformes) from the eastern coast of Korea. J Appl Ichthyol 32(1):160–162.	$0.0010 * \text{length}^{3.112}$
62	*length > wet*	Hegele-Drywa J, Normant M, Szwarc B, Podluska A (2014) Population structure, morphometry and individual condition of the non-native crab *Rhithropanopeus harrisii* (Gould, 1841), a recent coloniser of the Gulf of Gdańsk (southern Baltic Sea). Oceanologia 56(4):805–824.	$0.006 * \text{length}^{2.77}$
63	*length > wet*	Froese R, Thorson JT, Reyes RB (2014) A Bayesian approach for estimating length-weight relationships in fishes. J Appl Ichthyol 30(1):78–85.	$0.0138 * \text{length}^{3.14}$
64	*dry > wet*	Ricciardi A, Bourget E (1998) Weight-to-weight conversion factors for marine benthic macroinvertebrates. Mar Ecol Prog Ser 163:245–251.	(dry/20.0) * 100
65	*length > wet*	Froese R, Thorson JT, Reyes RB (2014) A Bayesian approach for estimating length-weight relationships in fishes. J Appl Ichthyol 30(1):78–85.	$0.00776 * \text{length}^{3.07}$
66	*length > wet*	Park KY, Oh CW (2002) Length-weight relationship of bivalves from coastal waters of Korea. Naga, ICLARM Q 25(1):21–22.	$(1.5119*10^{-4}) * \text{length}^{2.98}$

67	*length > wet*	Robinson LA, et al. (2010) Length–weight relationships of 216 North Sea benthic invertebrates and fish. J Mar Biol Assoc United Kingdom 90(1):95–104.	$-3.445 + 2.509 * \log_{10}(\text{length})$
68	*length > wet*	Rumohr, H., Brey, T. & Ankar, S. (1987) A compilation of biometric conversion factors for benthic invertebrates of the Baltic Sea. Baltic Marine Biology, 9, 1–56.	$0.0058 * \text{length}^{3.015}$
69	*length > wet*	Rumohr, H., Brey, T. & Ankar, S. (1987) A compilation of biometric conversion factors for benthic invertebrates of the Baltic Sea. Baltic Marine Biology, 9, 1–56.	$-1.249 + 2.662 * \log_{10}(\text{length})$
70	*length > wet*	Silva JF, Ellis JR, Ayers RA (2013) Cefas Science Series Technical Report no. 150. Length-weight relationships of marine fish collected from around the British Isles.	$0.0022 * \text{length}^{3.1194}$

2. SUPPLEMENTARY EXPERIMENTS

During 2015, consumers and resources were collected in the locality of Strangford Lough, Northern Ireland (54.48102° N, 5.58841° W) by using a combination of baited potting, hand diving, kick netting and digging intertidally (refer to Table S4 for specific methods used to collect each consumer and resource). Our approach here was to maximise data collection given the sizes and species we could obtain from the field. Predators were blotted dry with paper towels before being individually weighed and pre-sorted into size classes that provided sufficient numbers to use in experiments avoiding pseudoreplication. Outliers (i.e. very small and very large individuals) were returned to the sea. Prey (*Mytilus edulis* and *Enchinogammarus marinus*) were visually sorted by size and then subsampled (n = 30-50) and weighed to estimate prey sizes for each functional response (Table S4). Stock of each size-species combination was held separately in 1000 L flow-through aquariums at Queen's University Marine Laboratory, continuously supplied with sand-filtered seawater pumped from Strangford Lough. Temperatures averaged 11.14 °C (SD = 2.98 °C) over the period. Stock was fed *ad libitum* with either mixed size classes of mussels, gammarids, shredded marcroalgae, or with a microalgal suspension (*Tetraselmis suecica*) (as appropriate to the consumer or resource, refer to Table S4 for specific feeding regimes for each consumer and resource).

Depending on predator-prey size combinations, prey densities of 1, 2, 3, 4, 8, 10, 12, 15, 16, 20, 25, 30, 32, 40 and 64 individuals were used in experimental trials (n = 2-6 at each prey density) with higher densities being utilised for treatments where predators were not satiated (e.g. large crabs feeding on small mussels). Refer to Table S4 for the density and replication specifics of each functional response pair. Replication and densities used also reflect optimal use of available sizes and quantities of predators and prey from field collections. Trials were conducted in opaque grey cuboid mesocosms (refer to Table S1 for sizes and volumes) on outdoor seawater tables provisioned with flow-through sand-filtered seawater. Trials were initiated between 16:00 and 18:00 h on the introduction of single predators (starved for 24 h to standardise hunger) into mesocosms provisioned (1-3 h prior to predator introduction) with a block-randomised prey density treatment (where each functional response in Table S4 constitutes a single experimental block) and were terminated between 16 h and 3 d later on the removal of predators. Trial times reflect the varying efficacies of predator treatments, with, for example, large crabs quickly consuming dozens of small prey items, requiring relatively short trials, and starfish spending hours handling single prey items, requiring relatively long trials (Table S4). Controls were predator-free replicates (n = 3) of the highest and lowest prey densities used for each predator-prey combination in Table S4: prey survival was 100% in all controls, indicating that background mortality did not affect our results. Surviving prey were counted on the termination of trials and were not reused in experiments. On completion of trials, all surviving prey, and all predators, were returned to the sea.

Table S4 Details of predators (consumers) and prey (resources) used in experimental trials, including sizes (wet masses), collection methods, stock feeding regimes, trial prey densities and replication, trial temperatures and arena footprints (all interactions are 2D). Controls for each functional response (row) were predator free treatments of the highest and lowest prey densities used in that response (*n* = 3). Functional response trials were block randomised (where each row is one block).

arena footprint (cm^2)	trial temperature (°C)	trial time (days)	consumer	consumer mass (g)	field collection method	stock feeding regime	resource	field collection method	stock feeding regime	resource mass (g)	trial prey densities	*n*
407.64	7.91	1.00	*Carcinus maenas*	6.856	pot	gammarids	*E. marinus*	kick net	macroalgae	0.047	2, 4, 8, 16, 32	3
407.64	7.91	1.00	*Lipophrys pholis*	3.360	hand	gammarids	*E. marinus*	kick net	macroalgae	0.045	2, 4, 8, 16, 32	3
407.64	7.99	1.00	*Lipophrys pholis*	9.590	hand	gammarids	*E. marinus*	kick net	macroalgae	0.049	2, 4, 8, 16, 32	3
905.25	7.85	1.00	*Carcinus maenas*	25.800	pot	gammarids	*E. marinus*	kick net	macroalgae	0.055	2, 4, 8, 16, 32	3
407.64	8.03	1.00	*Pholis gunnellus*	5.705	hand	gammarids	*E. marinus*	kick net	macroalgae	0.050	2, 4, 8, 16	3
1970.25	8.00	0.66	*Carcinus maenas*	102.800	pot	mussels	*M. edulis*	dig	*T. suecica*	13.665	2, 4, 8, 16, 32	4
1970.25	8.00	0.66	*Carcinus maenas*	94.025	pot	mussels	*M. edulis*	dig	*T. suecica*	6.518	2, 4, 8, 16, 32	4
1970.25	8.00	0.66	*Carcinus maenas*	96.570	pot	mussels	*M. edulis*	dig	*T. suecica*	1.076	2, 4, 8, 16, 32, 64	4
1970.25	7.92	0.66	*Carcinus maenas*	66.863	pot	mussels	*M. edulis*	dig	*T. suecica*	13.665	2, 4, 8, 16, 32	4
1970.25	8.00	0.66	*Carcinus maenas*	60.170	pot	mussels	*M. edulis*	dig	*T. suecica*	6.518	2, 4, 8, 16, 32	4
1970.25	8.00	0.66	*Carcinus maenas*	60.235	pot	mussels	*M. edulis*	dig	*T. suecica*	1.076	2, 4, 8, 16, 32, 64	4
1970.25	7.92	0.66	*Carcinus maenas*	25.075	hand	mussels	*M. edulis*	dig	*T. suecica*	13.665	2, 4, 8, 16, 32	4
1970.25	8.00	0.66	*Carcinus maenas*	25.263	hand	mussels	*M. edulis*	dig	*T. suecica*	6.518	2, 4, 8, 16, 32	4
1970.25	8.00	0.66	*Carcinus maenas*	25.185	hand	mussels	*M. edulis*	dig	*T. suecica*	1.076	2, 4, 8, 16, 32, 64	4
905.25	12.00	1.00	*Actinia equina*	0.158	hand	gammarids	*E. marinus*	hand	macroalgae	0.122	2, 4, 6, 10, 15, 25	3
905.25	12.00	1.00	*Actinia equina*	1.674	hand	gammarids	*E. marinus*	hand	macroalgae	0.122	2, 4, 6, 10, 15, 25	3
905.25	12.00	1.00	*Actinia equina*	8.990	hand	gammarids	*E. marinus*	hand	macroalgae	0.122	2, 4, 6, 10, 15, 25	3
905.25	12.30	1.00	*Actinia equina*	1.674	hand	gammarids	*E. marinus*	hand	macroalgae	0.059	2, 4, 6, 10, 15, 25	3
905.25	12.30	1.00	*Actinia equina*	1.674	hand	gammarids	*E. marinus*	hand	macroalgae	0.008	2, 4, 6, 10, 15, 25	3
905.25	12.00	1.00	*Actinia equina*	0.158	hand	gammarids	*E. marinus*	hand	macroalgae	0.059	2, 4, 6, 10, 15, 25	3

905.25	11.90	1.00	*Actinia equina*	8.990	hand	gammarids	*E. marinus*	hand	macroalgae	0.059	2, 4, 6, 10, 15, 25	3
905.25	11.90	1.00	*Actinia equina*	0.158	hand	gammarids	*E. marinus*	hand	macroalgae	0.008	2, 4, 6, 10, 15, 25	3
4190	15.22	2.75	*Marthasterias glacialis*	547.885	dive	mussels	*M. edulis*	dig	*T. suecica*	13.665	1, 2, 3, 4, 5, 6	2-6
1970.25	15.02	2.75	*Asterias rubens*	98.720	dive	mussels	*M. edulis*	dig	*T. suecica*	5.704	1, 2, 3, 4, 5, 6	6
905.25	15.02	2.75	*Asterias rubens*	38.834	dive	mussels	*M. edulis*	dig	*T. suecica*	5.704	1, 2, 3, 4	5
1970.25	15.22	2.75	*Asterias rubens*	160.280	dive	mussels	*M. edulis*	dig	*T. suecica*	5.704	1, 2, 3, 4, 6, 8	2-5
1970.25	13.10	2.75	*Asterias rubens*	98.720	dive	mussels	*M. edulis*	dig	*T. suecica*	1.076	1, 2, 3, 4	2
1970.25	15.20	0.75	*Necora puber*	157.364	pot	mussels	*M. edulis*	dig	*T. suecica*	3.440	2, 4, 8, 12, 16, 20	4
1970.25	15.20	0.75	*Necora puber*	70.534	pot	mussels	*M. edulis*	dig	*T. suecica*	4.440	2, 4, 8, 12, 16, 20	5
1970.25	13.10	0.75	*Cancer pagurus*	205.611	pot	mussels	*M. edulis*	dig	*T. suecica*	4.440	2, 4, 8, 12, 16, 20, 30, 40	2-3
1970.25	13.50	0.75	*Cancer pagurus*	407.110	pot	mussels	*M. edulis*	dig	*T. suecica*	0.120	1, 2, 3, 4, 8, 12, 16, 20	2-5

3. MIXED EFFECTS MODELS

The following code establishes our maximal mixed effects model using `lme4` in R for either capture rates or maximum feeding rates as the response variable.

```
### maximal model syntax in lme4

maxmod<-lmer(log(response)~ # capture or maximum feeding rates
           # all 2-ways between metabolic predictors
           #(continuous):
             ((log(consumer_mass) + log(resource_mass) +
             log(temp))^2 +
           # non-interacting categorical predictor (3 levels):
             encounter_strategy) +
           # random effect structure:
             (1|taxonomic_group),
             data=d, # dataframe with required columns
             REML = FALSE, # REML = FALSE
             )
```

The object `maxmod` can be interrogated in the usual ways, for example `summary(maxmod)`, and passed to `dredge()` etc:

```
set<-dredge(maxmod) # create named object from dredge()

#95% confidence set:

model.avg(set, subset = cumsum(weight) <= .95) # get averaged
# coefficients from set(not used in this analysis)

summary(get.models(set, 1)[[1]])
```

Table S5 Complete fixed-effects model sets and AIC_c scores for both capture and maximum feeding rates as response variables given maximal model structure (`maxmod`). Numeric values indicate coefficients for each variable in the model, '+' indicates that the multilevel factor 'encounter strategy' was included in the model, '-' that the variable was not included in the model. '*' indicates an interaction between two named variables. Model with the lowest AICc value is highlighted in bold.

a) Capture rate models

intercept	encounter strategy	cons (g)	res (g)	temp °C	cons (g) * res (g)	cons (g) * temp °C	res (g) * temp °C	AICc
**-8.08**	+	**0.58**	**0.59**	**1.84**	**-**	**-**	**0.12**	**783.95**
-13.58	+	1.97	0.28	3.96	-0.02	-0.56	0.24	784.11
-6.98	+	0.54	0.91	1.48	-0.01	-	-	784.44
-6.64	+	0.66	0.87	1.21	-	-	-	784.63
-14.54	-	2.06	0.22	3.92	-0.02	-0.60	0.25	784.74
-10.43	+	1.23	0.31	2.75	-	-0.24	0.23	784.81
-7.85	+	0.54	0.70	1.80	0.00	-	0.09	785.68
-7.96	-	0.55	0.55	1.57	-	-	0.14	785.95
-6.75	-	0.50	0.92	1.18	-0.01	-	-	786.15
-7.75	+	0.88	0.91	1.81	-0.01	-0.13	-	786.16
-6.29	+	0.43	0.88	1.10	-	0.08	-	786.47
-9.87	-	1.08	0.31	2.32	-	-0.20	0.23	787.19
-7.74	-	0.50	0.68	1.53	-0.01	-	0.09	787.27
-9.49	-	1.10	0.87	1.97	-0.02	-0.22	-	787.35
-5.84	-	0.61	0.91	0.74	-	-	-	787.94
-3.98	-	0.60	0.91	-	-	-	-	788.30
-3.52	+	0.62	0.91	-	-	-	-	788.40
-5.51	-	0.23	0.91	0.60	-	0.13	-	788.92
-3.87	-	0.55	0.91	-	-0.01	-	-	789.27
-3.39	+	0.57	0.93	-	0.00	-	-	790.17
-7.85	+	-	0.34	2.54	-	-	0.39	824.88
-8.46	-	-	0.17	2.59	-	-	0.37	846.79
-0.92	+	-	1.42	-	-	-	-	849.82
-1.40	+	-	1.42	0.19	-	-	-	851.88
-17.17	+	2.17	-	3.20	-	-0.33	-	859.08
-15.86	+	1.25	-	2.66	-	-	-	861.08
-1.42	-	-	1.22	-	-	-	-	865.04
-22.16	-	2.12	-	3.13	-	-0.31	-	866.40
-2.85	-	-	1.21	0.51	-	-	-	866.45
-20.45	-	1.25	-	2.60	-	-	-	867.87
-9.27	+	1.22	-	-	-	-	-	876.93
-13.36	-	1.26	-	-	-	-	-	884.92
-13.59	+	-	-	1.80	-	-	-	1058.21
-8.79	+	-	-	-	-	-	-	1058.60
-21.84	-	-	-	2.78	-	-	-	1068.21
-14.28	-	-	-	-	-	-	-	1072.97

b) Maximum feeding rate models

intercept	encounter strategy	cons (g)	res (g)	temp °C	cons (g) * res (g)	cons (g) * temp °C	res (g) * temp °C	AICc
**-6.07**	**+**	**1.44**	**0.27**	**1.78**	**-**	**-0.39**	**-**	**657.16**
-7.33	-	1.38	0.32	1.87	-	-0.38	-	658.79
-7.71	+	1.71	0.05	2.42	-	-0.50	0.09	658.88
-6.16	+	1.48	0.27	1.82	0.00	-0.41	-	659.45
-9.06	-	1.69	0.07	2.62	-	-0.51	0.11	660.12
-7.42	-	1.44	0.33	1.94	0.00	-0.41	-	660.98
-4.03	+	0.45	0.26	0.98	0.01	-	-	661.11
-7.86	+	1.76	0.05	2.48	0.00	-0.52	0.09	661.20
-1.54	+	0.45	0.29	-	0.01	-	-	661.98
-9.23	-	1.77	0.07	2.74	0.00	-0.55	0.11	662.31
-4.68	-	0.38	0.36	1.03	0.01	-	-	662.72
-4.15	-	0.25	0.42	1.19	-	-	-	662.88
-4.41	+	0.29	0.32	1.25	-	-	-	662.93
-2.72	+	0.36	0.60	0.56	-	-	-0.12	662.95
-3.37	+	0.45	0.39	0.73	0.01	-	-0.05	663.10
-1.24	-	0.35	0.43	-	0.01	-	-	663.84
-3.38	-	0.29	0.61	0.75	-	-	-0.08	664.12
-8.86	+	1.88	-	2.35	-	-0.49	-	664.31
-4.43	-	0.39	0.43	0.89	0.01	-	-0.03	664.85
-0.46	-	0.20	0.47	-	-	-	-	665.72
-1.10	+	0.24	0.37	-	-	-	-	665.93
-12.97	-	1.97	-	2.69	-	-0.53	-	667.00
0.50	-	-	0.57	-	-	-	-	671.82
-1.48	-	-	0.57	0.74	-	-	-	671.91
-2.47	-	-	0.42	1.11	-	-	0.05	673.56
-0.08	+	-	0.56	-	-	-	-	673.97
-7.58	+	0.49	-	1.80	-	-	-	674.24
-1.56	+	-	0.55	0.60	-	-	-	674.95
-2.53	+	-	0.41	0.96	-	-	0.05	676.68
-10.75	-	0.49	-	2.03	-	-	-	678.23
-3.27	+	0.45	-	-	-	-	-	682.57
-5.28	-	0.47	-	-	-	-	-	690.87
-7.67	+	-	-	1.12	-	-	-	731.49
-3.67	+	-	-	-	-	-	-	732.25
-9.96	-	-	-	1.59	-	-	-	736.35
-5.67	-	-	-	-	-	-	-	740.54

4. AGENT-BASED MODEL PARAMETERISATION

Empirical data on the scaling of velocity (m s^{-1}) in aquatic organisms is provided by Vogel[1], covering unicellular flagellates and ciliates, invertebrates, fish, birds and mammals. Velocity is nearly proportional to body length throughout the whole range of terrestrial and aquatic running and swimming organisms, except for the very largest (approximately: >1-10 m body lengths)[2,3]. Because all organisms in our dataset (Table S1) are well below this threshold, we extracted all data points below 1 m body length and refitted via OLS in log-log space (Fig S1) to obtain:

$$V = 2.67 \cdot l^{1.05} \qquad \text{(S1)}$$

where V is consumer or resource velocity (m s^{-1}) and l is consumer or resource body length (m).

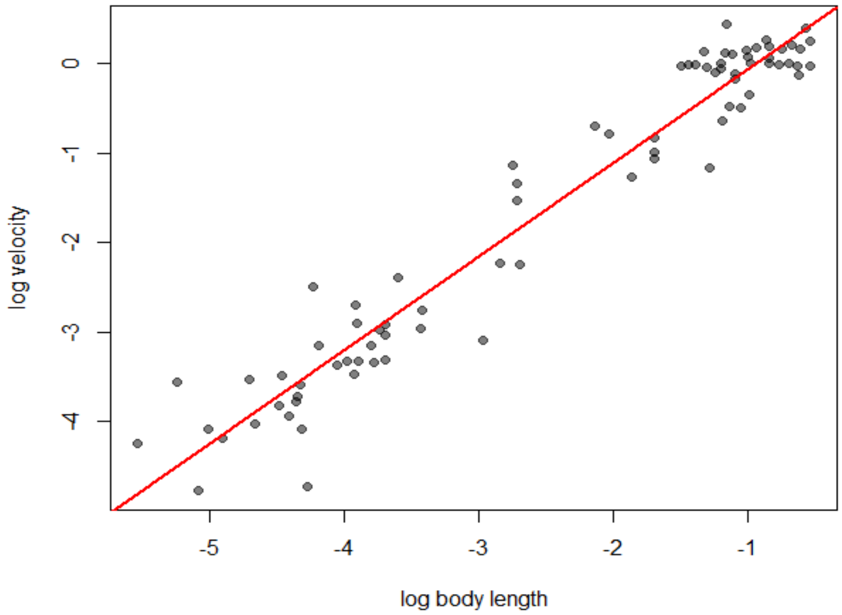

Fig S1 Velocity data extracted from Vogel[1]. Body lengths are in log m and velocities are in log m s^{-1}. Data refitted with OLS. Intercept ± s.e. = 0.983 ± 0.08***; slope ± s.e. = 1.05 ± 0.03***.

Consumer encounter regions are defined by reaction distances, which determines the radius of a 2D or 3D region surrounding them. Across feeding strategies and biomes, reaction distances scale with consumer mass as[4]:

$$D_g = 1.62 \cdot m^{0.36} \qquad \text{(S2)}$$

Where D_g is the generalised consumer reaction distance (m) and m is consumer mass (kg). Although this empirically well-supported relationship holds for active and passive encounter strategies (Fig 1), filter feeders do not form part of the data underpinning eqn S2[4] and might reasonably constitute an exception. Therefore, we also use the scaling of clearance rates (i.e. the volume of water cleared of resource per unit time) with consumer body mass (in Mytilids

clearance rates scale with $m^{0.47\text{-}0.72}$) to define an alternative encounter region and reaction distance for filter feeders using pooled exponents and constants from[5]:

$$D_f = 0.07 \cdot m^{0.21} \tag{S3}$$

Where D_f is the clearance-based reaction distance (m) and m is mass (kg). To demonstrate our findings are not sensitive to assumptions about the relative capacity of filter feeders to encounter resources in the environment, we parameterise our Agent-Based Model (ABM) with D_f, D_g and $2D_g$ (Fig S5).

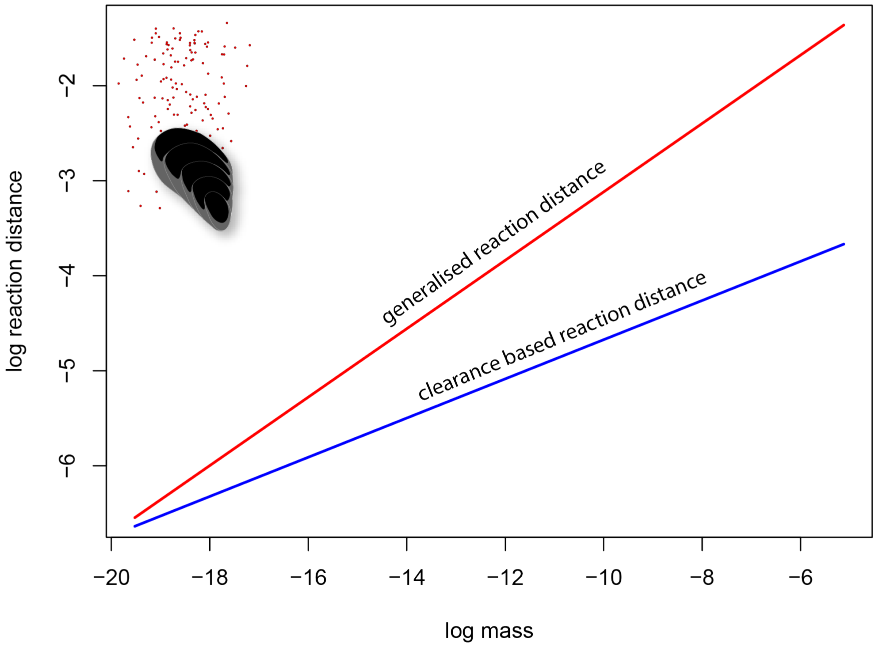

Fig S2 Predicted scaling of reaction distance (log m) with consumer mass (log kg) across the range of filter feeder body masses in the dataset (Table S1). Red is the generalised reaction distance (eqn S2, underpinned by data in[4]) and blue is the clearance-based reaction distance (eqn S3, underpinned by data in[5]).

Where resources are scarce, resource consumption is limited by resource encounters, rather than resource handling times—which limit consumption where resources are abundant—and well-designed functional response trials should experimentally manipulate resource density to capture these two extremes[6]. Broadly, resource density should scale negatively with resource mass (i.e. Damuth's Law[7]) whilst optimal resource size should scale positively with consumer size[8]. That is, larger consumers will search for larger but rarer resources in their environment. However, feeding strategy (Fig 1) resource type and ecological context[9] might all limit encounters. Because we are interested in encounter limitation—which occurs at low resource densities—we regressed resource size (Fig S3) and *minimum* resource density (Table S1) against consumer size (using OLS in log-log space) for each of the three feeding

strategies retained in the mixed-effects models to obtain strategy-specific relations (Figs S3-4; Table S6):

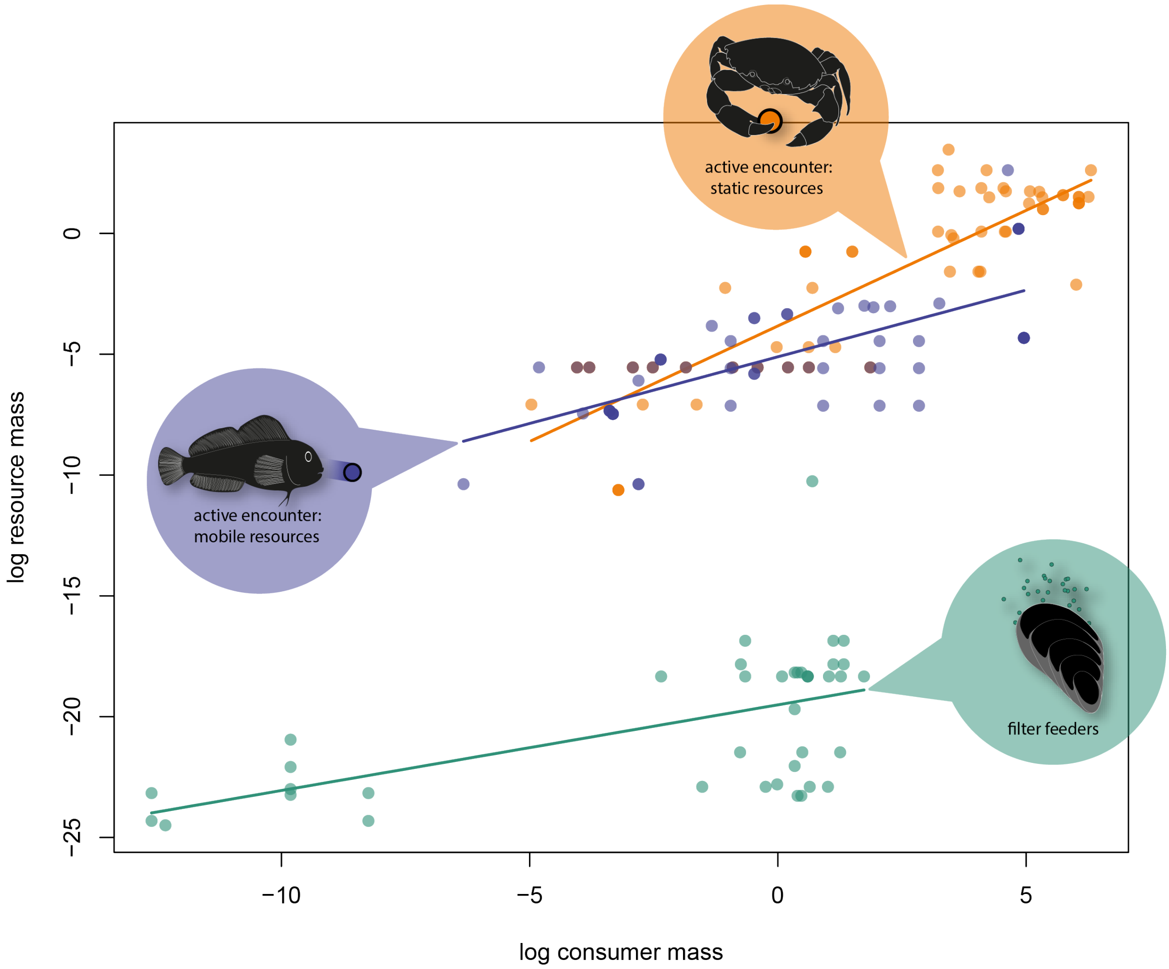

Fig S3 Scaling of resource mass (log g) with consumer mass (log g) for each of the resource encounter strategies parameterised in ABMs. Underlying data are in Table S1, fitted parameters are in Table S6.

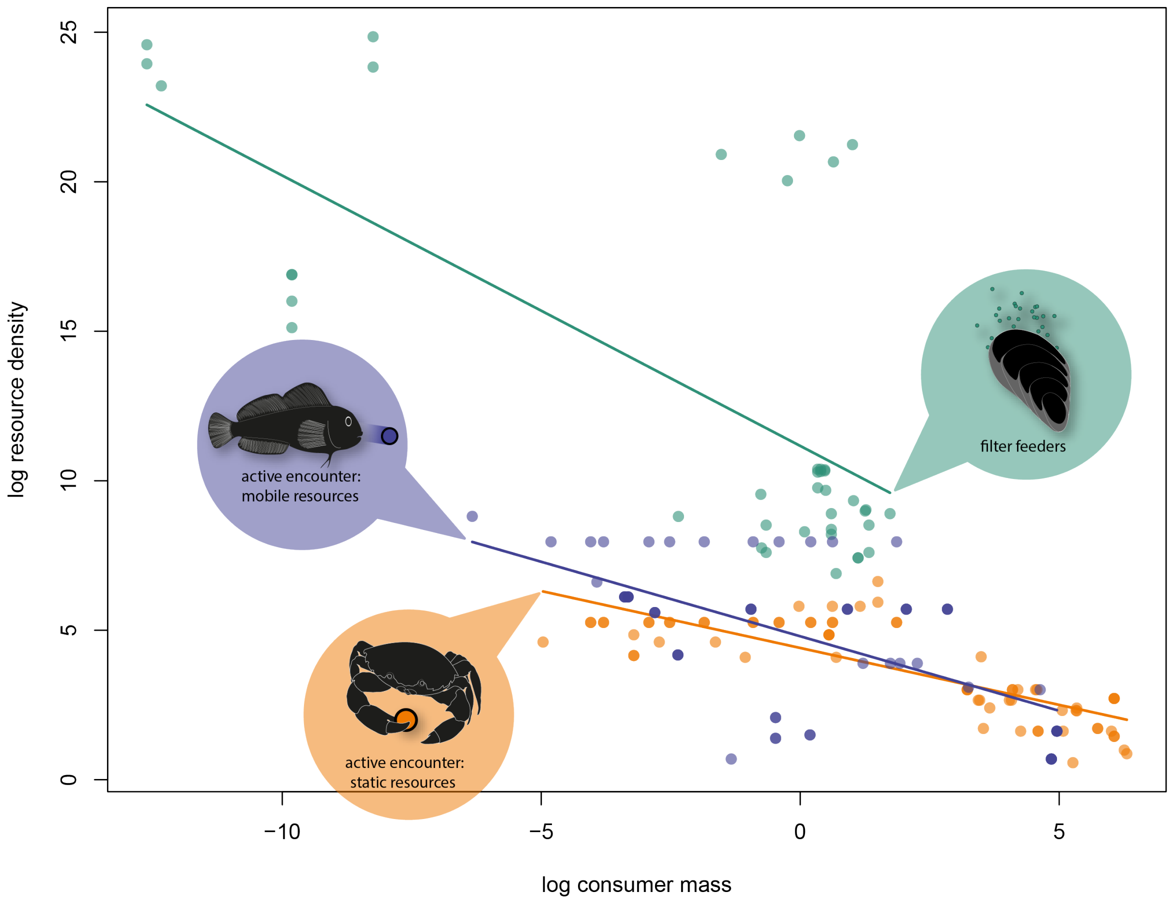

Fig S4 Scaling of resource density (log unitary individuals m$^{2-1}$) with consumer mass (log g) for each of the resource encounter strategies parameterised in ABMs. Underlying data are in Table S1, fitted parameters are in Table S4.

Table S6 OLS fits of log resource density (unitary individuals m$^{2-1}$ or m$^{3-1}$) and log resource size (g) on log consumer size (g) for each of the encounter strategies parameterised in ABMs.

strategy	resource density intercept	s.e.	resource density slope	s.e.	resource size intercept	s.e.	resource size slope	s.e.
active: mobile	4.80***	0.25	-0.50***	0.08	-5.11***	0.23	0.55***	0.08
active: static	4.41***	0.13	-0.38***	0.03	-3.83***	0.25	0.96***	0.07
filter feeder	11.17***	0.77	-0.9***	0.15	-19.51***	0.43	0.35***	0.09

Thus, for a given consumer size and encounter strategy, ABMs are parameterised with predicted encounter regions and resources of predicted size and density—with predicted velocities applied to consumers, resources, or both (as appropriate to encounter strategy). Additionally, we also make a simple but well-supported assumption that those consumers and resources that move do so via random walks (e.g.[10]) at mass-specific velocities (eqn S1). For planktonic unicells (i.e. the typical resource for filter feeders in our data) we impose an additional movement parameter onto the random walk: a unidirectional velocity of 0.1 m s^{-1} ± 0.01 parallel to the seabed to simulate a laminar flow. This figure is both environmentally-relevant and within the optimal range for filter feeder clearance rates[11,12]. We make no additional assumptions about friction between the water column and seabed because the

feeding rates of many of the filter feeders in our dataset are tested in either raised[13] or suspended[14] contexts, and we are concerned with testing encounter limitation given optimal conditions.

5. AGENT-BASED MODEL CODE

The following example model is designed to work in Netlogo 3D, but principles are largely identical for 2D and 3D Netlogo models. The following two code blocks establish a 3D world with a single passive encounter consumer (for example a filter feeder or obligate sit-and-wait predator) on the seabed and 1000 resources randomly distributed in the water around it:

```
;;; This 3D world (above the mud) is approx 11*11*11 patches and represents
1 m^3 of water above a seabed

globals [encounters ;;; tracking encounters (cumulative)
  encounter-rate ;;;; rate of encounters
  sizetrack ;;;; a global mirroring the size of the consumer, which other
agentsets can call to redefine their size/abundance/speed/detection regions
after 1000 ticks
  birthtrack ;;; to track a set of size-abundance-speed-detection region
combinations for 1000 ticks before resetting with the next set
  ]

breed[consumers consumer]
breed [resources resource]
breed [Ds D] ;;; detection region of consumer

to setup
  clear-all
  reset-ticks
  set birthtrack 0
  set sizetrack 0.2 ;;; starting size
  set encounters 0
  ask patches
    with [ pzcor < 0 and pzcor > -9.5 ] ;;; below centre line of world:
consumer and bounce routine work above it
      [ set pcolor [218 160 62]]
   ask patches
    with [ pzcor = 0]
      [ set pcolor [250 160 62 50]]
   ask patches
    with [pzcor < -9.5]
      [ set pcolor [200 160 62]]
  setup-turtles ;;; this must come after patches in setup
end
```

```
to setup-turtles
  create-Ds 1 [set color [255 255 0 100]
  set shape "circle"
  set size 5 ; the actual size of the detection region (empirically
determined in specific models)
  ]
  ask Ds [setxyz 0 0 0.5]

  create-consumers 1 [set color red
  set shape "circle"
  set size 2] ;;; the min diameter of the agent to start (empirically
determined in specific models)
  ask consumers [setxyz 0 0 0.5]

  create-resources 1000 [set color white ;; the min resource abundance
(empirically determined in specific models)
  set shape "circle"
  set size 0.1]
  ask resources [setxyz random-xcor random-ycor (random-float(max-pzcor -
0.5)+ 0.5)]


end
```

The following code establishes movement via random walks and unidirectional currents as required, and provides a means to count consumer-resource encounters. An optional `grow` routine can be developed for examining scaling relationships by redefining consumer size, resource size, encounter regions, velocities as required using the globals `sizetrack` and `birthtrack` to reset parameters after the desired number of `ticks`:

```
to go
  move-resources ;;; to move resources, consumers or both (add move-
consumers argument as required)
  encounter ;;; count contacts
  tick
  ;; grow ;;; optional routine for scaling
  if ticks = 30000 [stop]  ;;; if using grow, this should be set to allow
each size class of consumer run for, e.g., 1000 ticks
end
```

```
to move-resources
ask resources [
  right random 90 ;;; simulated random walk
  left random 90
  tilt-up random 90
  tilt-down random 90
  fd ((2.674306) * (((size * 9.090909) / 100) ^ 1.04692) * 100)/ 9.090909
;; speed scaling
  ;;; simulated current in one direction:
  set heading 90
  set pitch 0
  set roll 0
  fd 0.1 * 11 ;; current speed ;;; 11 patches represent 1 m, so speed in
m/s * 11 = number of patches to go fd by
    ;; introduce uniform variance if required using random float

    while [ any? patches in-radius 1 with [pcolor = [218 160 62]] ]  [
    let nearest-patch min-one-of (patches with [pcolor = [218 160
62]])[distance myself] ;;; find the closest sandy patch
    face nearest-patch ;; face that patch
    set pitch pitch - 180 ;; face away from that patch
    fd 0.1 ;; move away from that patch
      ;; this should keep agents off the seabed
  ]
]
end


to encounter
  ask consumers [
    if any? resources in-radius ((size / 2) + ((((10 ^ 0.21) * ((((4 / 3)*
pi) * (((size * 9.090909) / 2) ^ 3) / 1000) ^ 0.36)) * 100) / 9.090909))
;;;; this is critical to give the consumer a detection region that relates
to it's *visual* size (in units of patches)
    [set encounters encounters + count resources in-radius ((size / 2) +
((((10 ^ 0.21) * ((((4 / 3)* pi) * (((size * 9.090909) / 2) ^ 3) / 1000) ^
0.36)) * 100) / 9.090909))
    set encounter-rate count resources in-radius ((size / 2) + ((((10 ^
0.21) * ((((4 / 3)* pi) * (((size * 9.090909) / 2) ^ 3) / 1000) ^ 0.36)) *
100) / 9.090909))] ;;;;;
  ]
end
```

6. FILTER FEEDER DETECTION REGION SENSITIVITY ANALYSIS

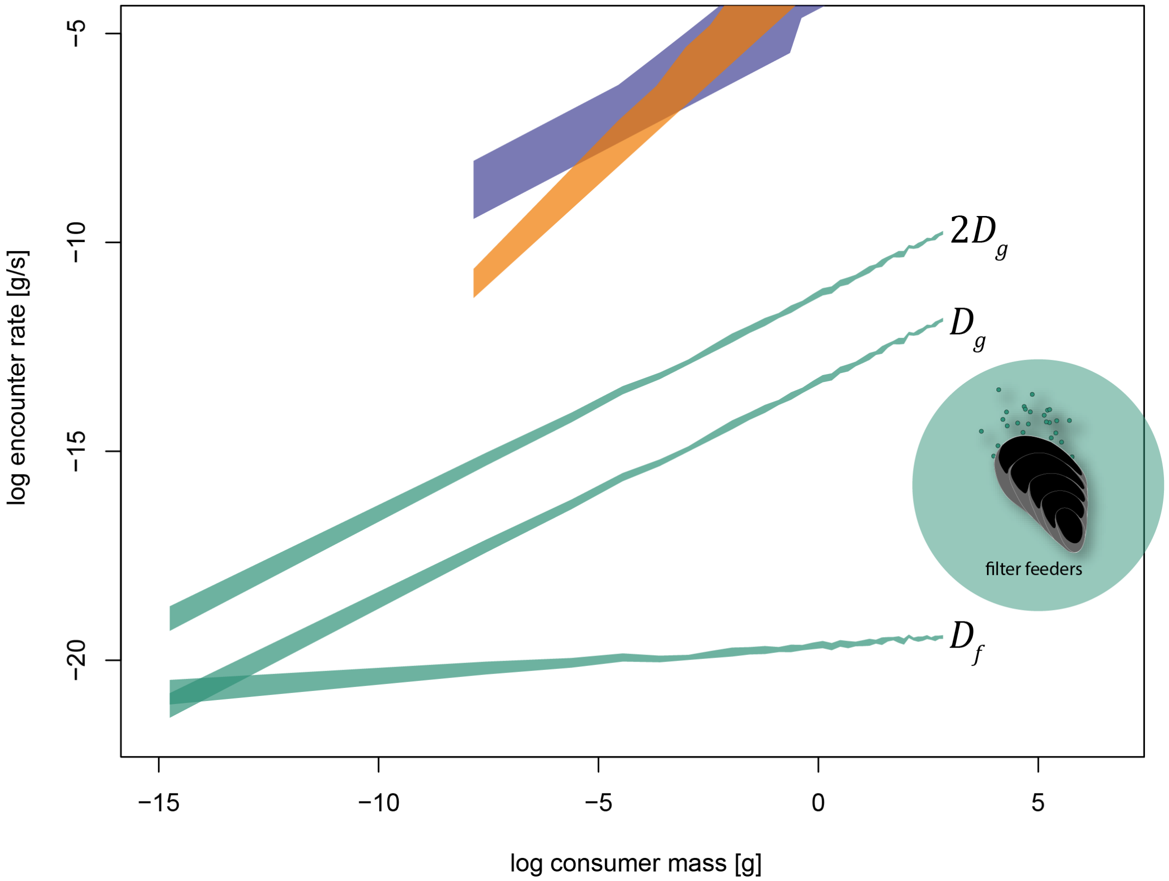

Figure S5 Agent based model outputs of resource encounter rates (biomass in g s^{-1}) among encounter strategies (see Fig 5 for full plot with active-mobile and active-static groups plotted entirely). Shaded areas are interpolated 0.025 (lower) and 0.975 (upper) quantiles (n = 1000) of encounters along the consumer mass axis. The model is empirically parameterised with respect to consumer masses, resource masses and resource densities (Figs S3 and S4) underpinning the meta-analysis (Figs 3 and 4). ABMs for filter feeders are parameterised with $2D_g$, D_g and D_f (see ABM parameterisation section for details).

References

1. Vogel, S. Modes and scaling in aquatic locomotion. *Integr. Comp. Biol.* **48**, 702–712 (2008).
2. Meyer-Vernet, N. & Rospars, J.P. How fast do living organisms move: Maximum speeds from bacteria to elephants and whales. *Am. J. Phys.* **83**, 719–722 (2015).
3. Hirt, M. R., Jetz, W., Rall, B. C. & Brose, U. A general scaling law reveals why the largest animals are not the fastest. *Nat. Ecol. Evol.* **1**, 1116–1122 (2017).
4. Pawar, S., Dell, A. I. & Savage, V. M. Dimensionality of consumer search space drives trophic interaction strengths. *Nature* **486**, 485–489 (2012).
5. Filgueira, R., Labarta, U. & Fernández-Reiriz, M. J. Effect of condition index on allometric relationships of clearance rate in *Mytilus galloprovincialis* Lamarck, 1819. *Rev. Biol. Mar. Oceanogr.* **43**, 391–398 (2008).
6. Pritchard, D. W., Paterson, R. A., Bovy, H. C. & Barrios-O'Neill, D. frair: An R package for fitting and comparing consumer functional responses. *Methods Ecol. Evol.* (2017). doi:10.1111/2041-210X.12784
7. Damuth, J. Population density and body size in mammals. *Nature* **290**, 699–700 (1981).
8. Barrios-O'Neill, D. *et al.* On the context-dependent scaling of consumer feeding rates. *Ecol. Lett.* **19**, 668–678 (2016).
9. Rizzuto, M., Carbone, C. & Pawar, S. Foraging constraints reverse the scaling of activity time in carnivores. *Nat. Ecol. Evol.* **2**, 247–253 (2018).
10. Bartumeus, F., Da Luz, M. G. E., Viswanathan, G. M. & Catalan, J. Animal search strategies: a quantitative random-walk analysis. *Ecology* **86**, 3078–3087 (2005).
11. Widdows, J., Lucas, J. S., Brinsley, M. D., Salkeld, P. N. & Staff, F. J. Investigation of the effects of current velocity on mussel feeding and mussel bed stability using an annular flume. *Helgol. Mar. Res.* **56**, 3–12 (2002).
12. Ackerman, J. D. Effect of velocity on the filter feeding of dreissenid mussels (*Dreissena polymorpha* and *Dreissena bugensis*): implications for trophic dynamics. *Can. J. Fish. Aquat. Sci.* **56**, 1551–1561 (1999).
13. Petersen, J. K., Schou, O. & Thor, P. Growth and energetics in ascidian *Ciona intestinalis*. *Mar. Ecol. Prog. Ser.* **120**, 175–184 (1995).
14. Bullivant, J. S. The rate of feeding of the bryozoan, *Zoobotryon verticillatum*. *New Zeal. J. Mar. Freshw. Res.* **2**, 111–134 (1968).
